# EPI-001 is a selective peroxisome proliferator-activated receptor-gamma modulator with inhibitory effects on androgen receptor expression and activity in prostate cancer

**DOI:** 10.18632/oncotarget.2924

**Published:** 2015-02-11

**Authors:** Lucas J. Brand, Margaret E. Olson, Preethi Ravindranathan, Hong Guo, Aaron M. Kempema, Timothy E. Andrews, Xiaoli Chen, Ganesh V. Raj, Daniel A. Harki, Scott M. Dehm

**Affiliations:** ^1^ Graduate Program in Microbiology, Immunology, and Cancer Biology, University of Minnesota, Minneapolis, MN, USA; ^2^ Masonic Cancer Center, University of Minnesota, Minneapolis, MN, USA; ^3^ Department of Medicinal Chemistry, University of Minnesota, Minneapolis, MN, USA; ^4^ Department of Urology, University of Texas Southwestern Medical Center, Dallas, Texas, USA; ^5^ Department of Food Science and Nutrition, University of Minnesota, Minneapolis, MN, USA; ^6^ Department of Laboratory Medicine and Pathology, University of Minnesota, Minneapolis, MN, USA

**Keywords:** EPI-001, Bisphenol A Diglycidyl Ether, Androgen Receptor, Prostate Cancer, Peroxisome Proliferator-Activated Receptor-gamma

## Abstract

The androgen receptor (AR) is a driver of prostate cancer (PCa) cell growth and disease progression. Therapies for advanced PCa exploit AR dependence by blocking the production or action of androgens, but these interventions inevitably fail via multiple mechanisms including mutation or deletion of the AR ligand binding domain (LBD). Thus, the development of new inhibitors which act through non-LBD interfaces is an unmet clinical need. EPI-001 is a bisphenol A-derived compound shown to bind covalently and inhibit the AR NH_2_-terminal domain (NTD). Here, we demonstrate that EPI-001 has general thiol alkylating activity, resulting in multilevel inhibitory effects on AR in PCa cell lines and tissues. At least one secondary mechanism of action associated with AR inhibition was found to be selective modulation of peroxisome proliferator activated receptor-gamma (PPARγ). These multi-level effects of EPI-001 resulted in inhibition of transcriptional activation units (TAUs) 1 and 5 of the AR NTD, and reduced AR expression. EPI-001 inhibited growth of AR-positive and AR-negative PCa cell lines, with the highest sensitivity observed in LNCaP cells. Overall, this study provides new mechanistic insights to the chemical biology of EPI-001, and raises key issues regarding the use of covalent inhibitors of the intrinsically unstructured AR NTD.

## INTRODUCTION

Prostate cancer (PCa) is the most commonly diagnosed male cancer in the US with approximately 233,000 new cases and 30,000 deaths predicted in 2014 [1]. Normal and cancerous prostate tissues are dependent on activation of the androgen receptor (AR) to support cell proliferation and survival [2, 3]. Thus, inhibiting AR activation serves as the basis for treating metastatic disease [4]. However, these therapies ultimately fail via a variety of molecular mechanisms [5]. Importantly, castration-resistant PCa (CRPC) tumors remain AR-dependent, as evidenced by the increased overall survival of patients treated with the second-generation androgen deprivation therapies enzalutamide [6–8] and abiraterone [9]. Despite these advances, resistance to enzalutamide and abiraterone is frequent and several AR re-activation mechanisms have been reported as likely drivers [10–14]. Therefore, development of novel AR-targeted therapeutics that are active in CRPC remains an important area of investigation [15].

The AR is a modular steroid hormone receptor transcription factor with the primary transcriptional activation function mapping to Transcriptional Activation Units (TAU)1 and TAU5 in the intrinsically unstructured AR NH_2_-terminal domain (NTD) [16, 17]. The functional importance of these domains is evidenced by the expression of AR splice variant proteins in CRPC, which are constitutively active AR species composed of the AR NTD and central DNA binding domain (DBD), but lacking the regulatory LBD [18, 19]. This highlights the clinical need for new therapeutics that exert action through non-LBD interfaces on the AR protein [18, 19]. EPI-001, a Bisphenol A diglicycyl ether (BADGE) derivative, was identified as a specific inhibitor of the AR that bound covalently to an undetermined structural motif in the AR NTD and inhibited the growth of androgen sensitive PCa and CRPC cells *in vitro* and *in vivo* [20, 21]. Here, we interrogated the mechanism by which EPI-001 inhibits the AR NTD. We show that EPI-001 is a general thiol modifier with myriad effects on AR expression and activity, and selectively modulates peroxisome proliferator-activated receptor-gamma (PPARγ) activity. Overall, this study provides novel insights to EPI-001 chemical biology that will be critical for ongoing development of AR NTD inhibitors.

## RESULTS

### EPI-001 inhibits transcriptional activity of both AR TAU1 and TAU5

**Figure 1:**
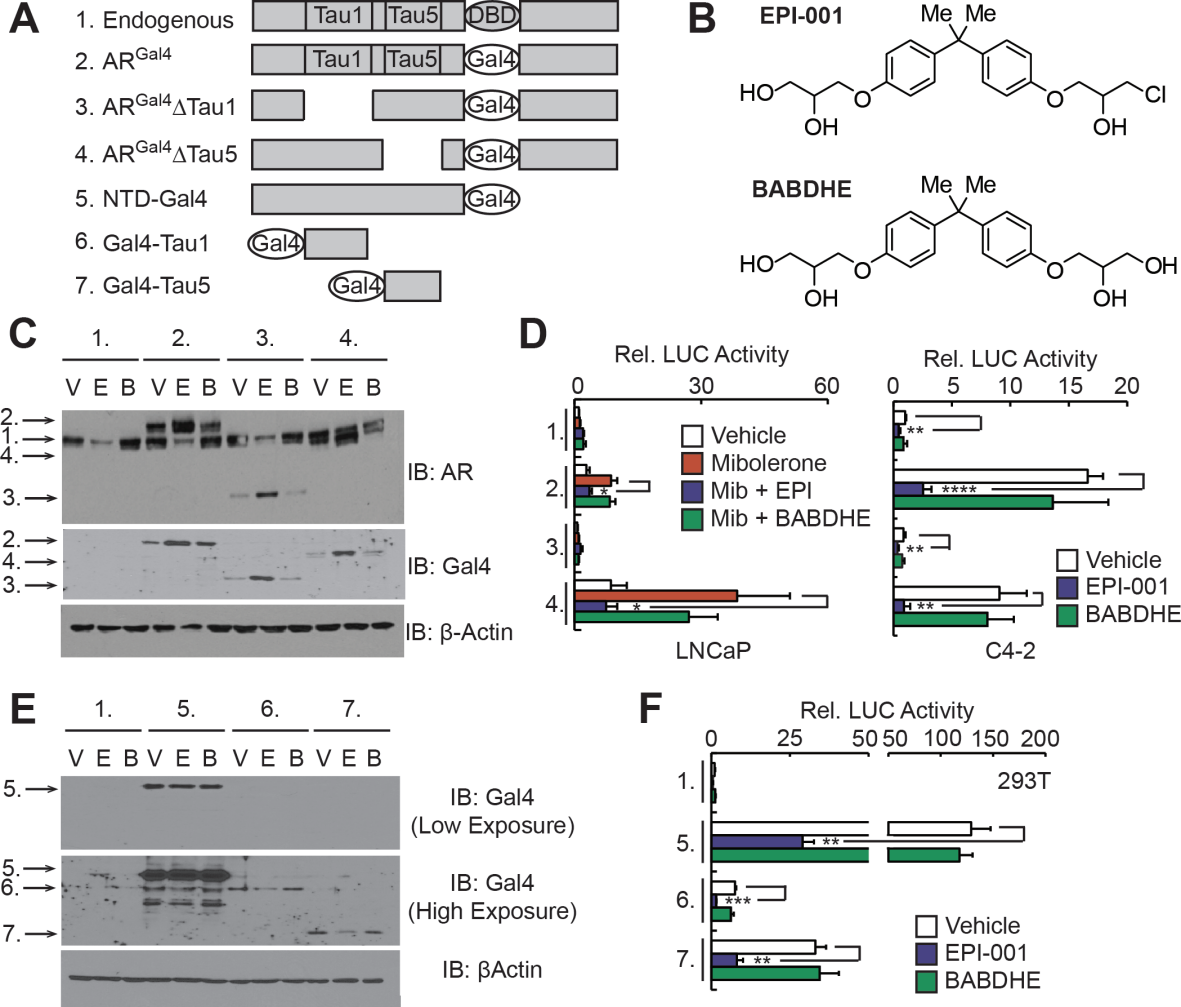
EPI-001 inhibits transcriptional activity of AR TAU1 and TAU5 domains in reporter-based assays **(A)** Schematic of Gal4-based AR expression constructs. **(B)** Chemical structures of EPI-001 and BABDHE. **(C and D)** LNCaP and C4-2 cells were transfected with constructs shown in panel *A* along with sPSA^Gal4^-luciferase and treated as indicated (V: Vehicle control; E: EPI-001 50 μM; B: BABDHE 50 μM). (*C*) LNCaP lysates treated in the absence of serum and androgen were subjected to western blot. (*D*) LNCaP and C4-2 protein lysates were subjected to luciferase assay. Bars depict mean +/− standard error (C4-2: *n* = 4 from 2 independent duplicate experiments; LNCaP: *n* = 5 from 2 independent duplicate/triplicate experiments). **(E and F)** 293T cells were transfected with the constructs shown in panel *A* along with pG5-luciferase and treated with the indicated drugs. Protein lysates were subjected to (*E*) western blot or (*F*) luciferase assay. Bars depict mean +/− standard error (*n* = 6 from 2 independent triplicate experiments). *p < 0.05, **p < 0.01, ***p < 0.001, ****p < 0.0001.

LNCaP cells were treated with a range of EPI-001 concentrations to identify doses that effectively inhibited AR-responsive luciferase reporters. Contrary to previous reports showing that 10 μM EPI-001 achieved robust AR inhibition [20], we observed that a 50 μM dose was required (Supplementary Figure S4). To identify the specific AR TAU through which 50 μM EPI-001 inhibited AR activity, we performed promoter tethering assays with an AR^Gal4^ hybrid wherein the AR DBD had been replaced with the yeast Gal4 DBD (Figure 1A, construct 2). As a negative control, we used bisphenol A bis [2,3-dihydroxypropyl] ether (BABDHE), as it is structurally similar to EPI-001 but contains a diol instead of a reactive chlorohydrin (Figure 1B) [21]. EPI-001 inhibited ligand-dependent AR^Gal4^ transcriptional activity in LNCaP cells (Figures 1C and 1D), as well as aberrant, ligand-independent ARGal4 transcriptional activity in the CRPC C4-2 cell line (Figure 1D). Deletion of TAU5 from AR^Gal4^ increased androgen-dependent AR^Gal4^ activity and decreased androgen-independent AR^Gal4^ activity, consistent with previous reports [22], but this deletion did not affect responsiveness to EPI-001 (Figure 1D). Conversely, deletion of TAU1 decreased androgen-dependent and –independent modes of AR^Gal4^ transcriptional activity in LNCaP and C4-2 cells (Figure 1D). This precluded evaluation of EPI-001 effects on TAU1 in LNCaP, but residual androgen-independent AR^Gal4^ transcriptional activity in C4-2 cells remained responsive to EPI-001 (Figure 1D). To test the responsiveness of discrete AR TAUs to EPI-001 directly, we tethered the entire AR NTD, or TAU1 or TAU5 fragments to the Gal4 DBD (Figure 1B, constructs 5–7). In all cell lines tested, EPI-001 inhibited transcriptional activity of the NTD-Gal4 hybrid (Figures 1E, 1F, and Supplementary Figure S5). The Gal4-TAU1 and Gal4-TAU5 fusion proteins displayed cell line-specific transcriptional activity, likely due to inefficient expression in PCa cell lines (Figures 1E, 1F, and Supplementary Figure S5). In 293T fibroblasts, transcriptional activity of the Gal4-TAU1 and –TAU5 constructs was potently inhibited by EPI-001 (Figures 1E and 1F). These data agree with previous reports of direct AR inhibition by EPI-001, but extend this knowledge by demonstrating the effects could not be mapped to a discrete AR TAU. This indicates two possible scenarios: 1) EPI-001 binds specifically to both TAU1 and TAU5, or 2) EPI-001 has a more general effect on transcriptional activities of TAU1 and TAU5.

### EPI-001 inhibits endogenous AR mRNA and protein expression

**Figure 2:**
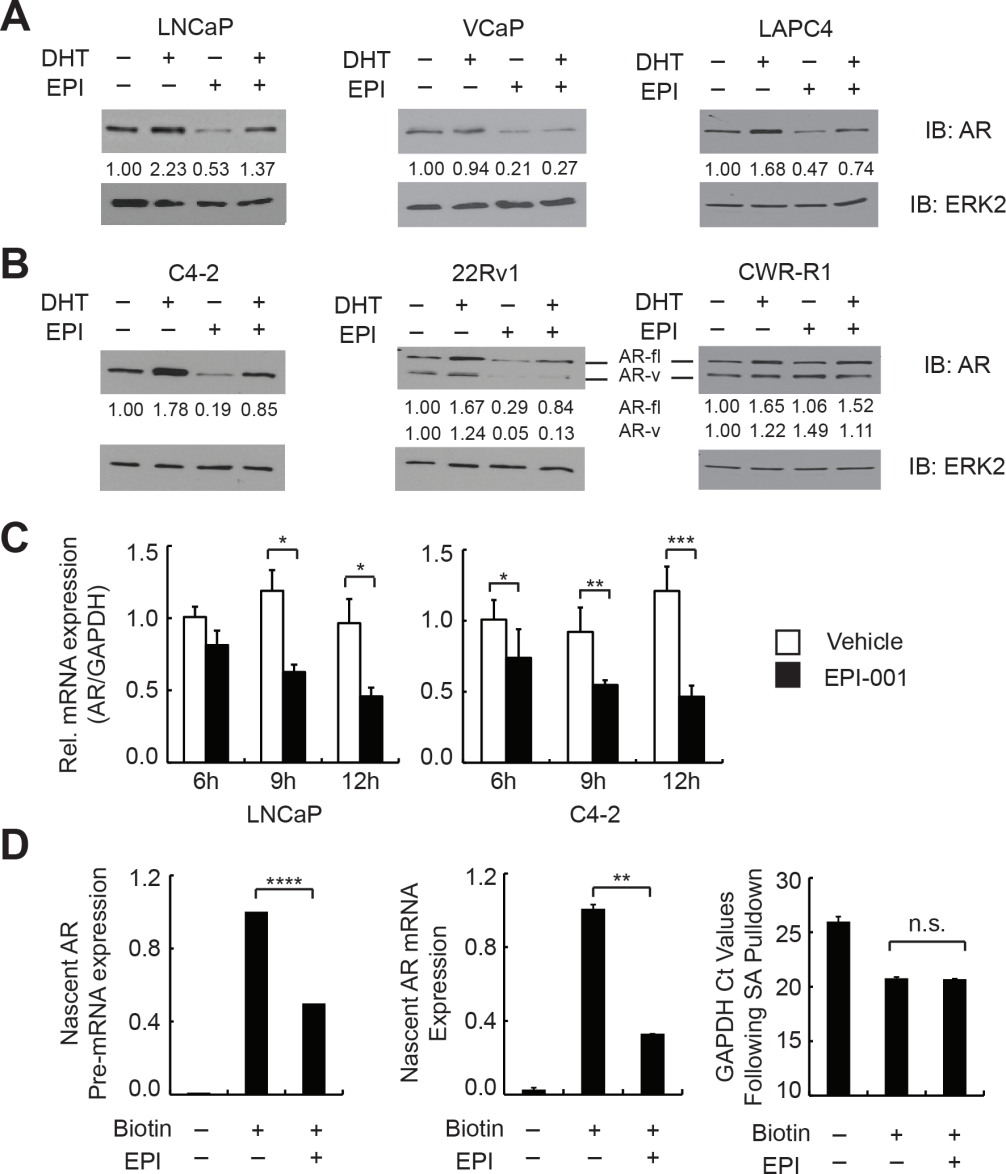
EPI-001 inhibits endogenous AR expression at the mRNA level **(A)** Androgen sensitive PCa and **(B)** CRPC cell lines were treated overnight in serum-free medium with 1 nM DHT and/or 50 μM EPI-001 as indicated, and analyzed by western blot. Densitometry data for both full length (AR-fl) and truncated variant (AR-v) isoforms are provided. **(C)** AR mRNA expression was analyzed by qRT-PCR at indicated time-points in LNCaP (*left*) and C4-2 (*right*) cells treated with 50 μM EPI-001. **(D)** LNCaP cells were treated with 50 μM EPI-001 or vehicle control for 8 h in serum free medium. Nascent transcripts were isolated and subjected to qRT-PCR using primers for AR pre-mRNA (Exon 1 FW & Intron 1 RV) or spliced mRNA (Exon 1 FW & Exon 2 RV). Bars depict mean +/− standard deviation (*C*: *n* = 3 from a triplicate experiment representative of two biological replicates; *D*: *n* = 6 from two biological replicates performed in triplicate). *P < 0.05; **P < 0.01; ***P < 0.001; ****P < 0.0001.

Interestingly, we observed that endogenous AR protein levels were consistently repressed in PCa cell lines treated with EPI-001 (Figure 1C). To explore this phenomenon, we tested the effect of EPI-001 on AR protein levels in a panel of androgen sensitive PCa (Figure 2A) and CRPC (Figure 2B) cell lines. In these cell lines, EPI-001 treatment decreased expression of full-length AR protein to varying degrees (Figures 2A and 2B). AR protein loss occurred between 8 and 16 hours of treatment and was independent of the proteasome (Supplementary Figure S6). In line with this, AR mRNA expression in LNCaP and C4-2 cells was reduced in response to EPI-001 at time points preceding the observed decreases in AR protein expression (Figure 2C). EPI-001 also inhibited the mRNA expression of AR and the AR target gene PSA in LAPC4 cells (Supplementary Figure S7A). EPI-001 treatment also decreased expression of truncated AR variant (AR-v) proteins expressed in the CRPC 22Rv1 cell line (Figure 2B). Interestingly, AR mRNA (Supplementary Figure S7B) and protein expression (Figure 2B) in CWR-R1 cells did not respond to EPI-001, nor did EPI-001 inhibit the expression of the AR target gene FKBP5 (Supplementary Figure S7B).

To test if the effects of EPI-001 on AR expression were due to decreased AR mRNA stability, we treated LNCaP with Actinomycin D alone or in combination with EPI-001. Treatment with EPI-001 did not accelerate AR mRNA decay following transcriptional blockade with Actinomycin D (Supplementary Figure S8). Consistent with this, we found that 50 μM EPI-001 reduced the rate of nascent AR mRNA synthesis in LNCaP cells (Figure 2D). Collectively, these data demonstrate that EPI-001 inhibits transcription of the AR gene.

### Inhibition of AR expression correlates with reduced cell growth in PCa and CRPC cell lines

**Figure 3:**
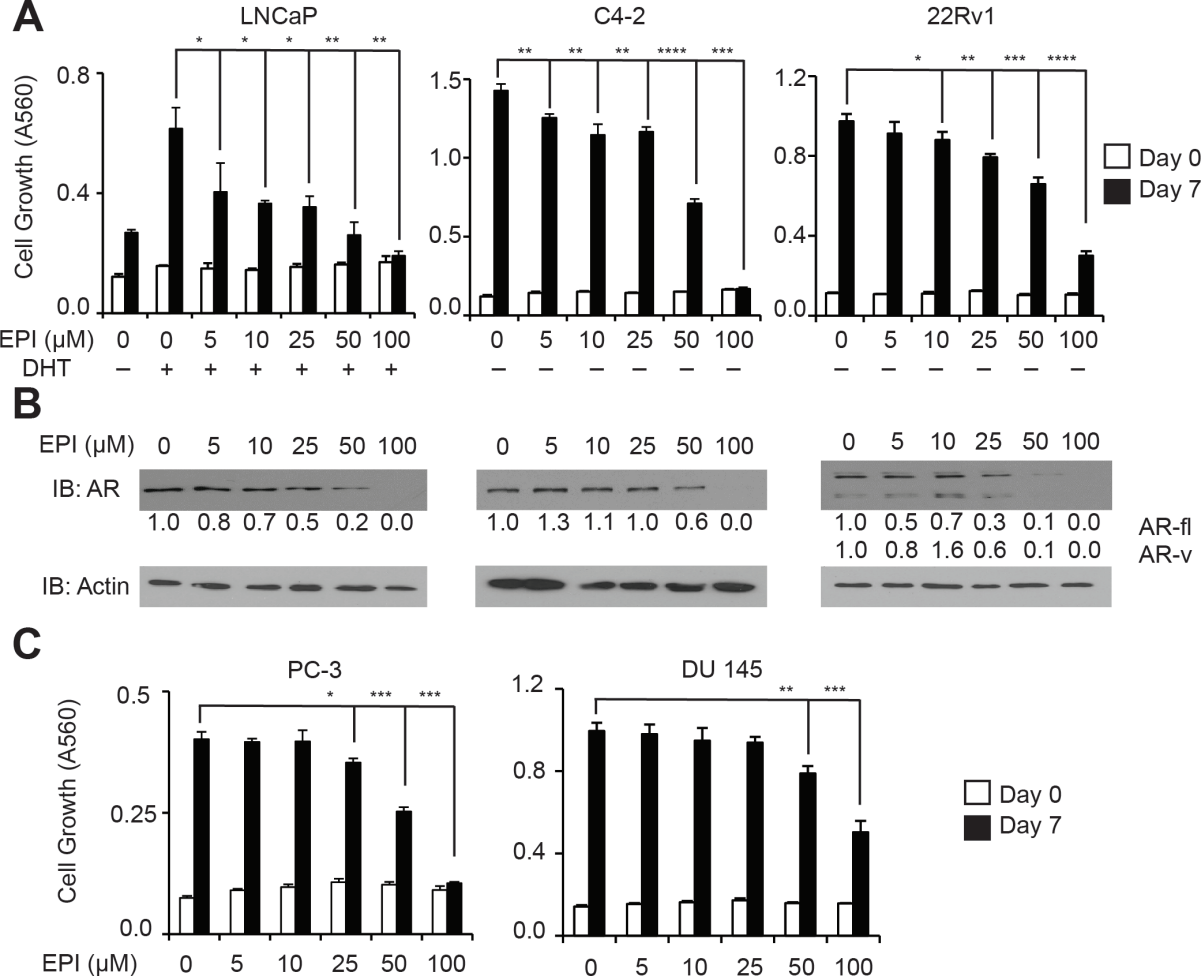
Dose-dependent inhibition of AR expression and PCa/CRPC cell growth mediated by EPI-001 **(A)** LNCaP, C4-2, and 22Rv1 cells were treated for 7 days in steroid-depleted medium containing 1 nM DHT and/or EPI-001 as indicated. Growth was monitored by crystal violet staining. Bars depict mean +/− standard deviation (*n* = 3 from a triplicate experiment representative of two biological replicates). **(B)** LNCaP, C4-2, and 22Rv1 cells were treated for 24 hours in serum free medium as in (*A*) and subjected to western blot. Densitometry data are provided. **(C)** AR-negative PC-3 and DU 145 cells were treated with EPI-001 and analyzed for growth exactly as in *A*. Bars depict mean +/− standard deviation (*n* = 3 from a triplicate experiment representative of two biological replicates). *P < 0.05; **P < 0.01, ***P < 0.001, ****P < 0.0001.

Based on these findings, we reasoned that inhibition of AR synthesis could be an important component of the EPI-001 anti-AR mechanism. EPI-001 inhibited growth of LNCaP cells at low concentrations, but in all other PCa cell lines, the concentrations at which EPI-001 inhibited growth (Figure 3A, Supplementary Figure S9) were the same concentrations that inhibited expression of AR or AR-V protein levels (Figure 3B). BABDHE also inhibited PCa and CRPC growth and AR expression, although higher doses were required than for EPI-001 (Supplementary Figure S10A & 10B), indicating the EPI-001 chlorohydrin moiety is important for inhibition of AR expression. Surprisingly, EPI-001 also inhibited growth of AR-negative PC-3 and DU 145 cell lines (Figure 3C), as well as the T47D breast carcinoma cell line (Supplementary Figures S11A and 11B). In T47D, biphasic modulation of AR as well as estrogen and progesterone receptors (ER and PR) was observed (Supplementary Figure S11C). These data signify AR-independent effects of EPI-001 in multiple cell types.

### EPI-001 action in PCa cells is similar to the PPARγ agonist, troglitazone

**Figure 4:**
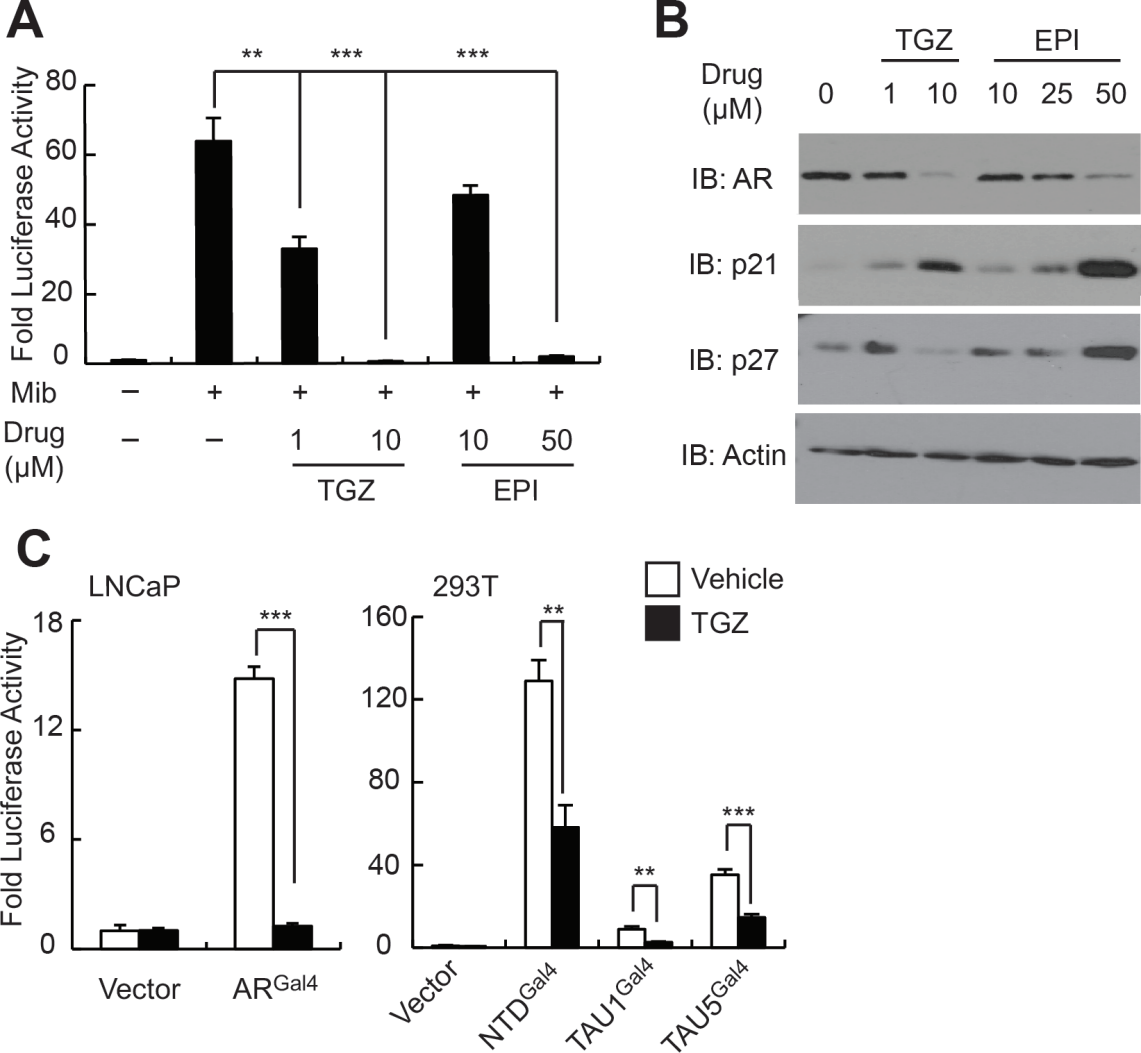
EPI-001 and the PPAR-γ agonist troglitazone have similar effects on AR expression and transcriptional activity **(A)** LNCaP cells were transfected with sPSA-Luciferase, treated with troglitazone (TGZ) or EPI-001 as indicated, and subjected to luciferase assay. **(B)** LNCaP cells were treated with troglitazone (TGZ) or EPI-001 as indicated and subjected to western blot. **(C)** LNCaP and 293T were transfected with the indicated Gal4-tethered AR constructs and either sPSA^Gal4^-Luciferase or pG5-Luciferase, respectively, and treated with 10 μM troglitazone or vehicle control as indicated. Bars represent mean +/− standard error (*n* = 6 samples from two independent triplicate experiments). *P < 0.05; **P < 0.01, ***P < 0.001.

Bisphenol A Diglycidyl Ether (BADGE), which is related structurally to EPI-001 but contains a bis-epoxide, has been shown to act as a selective PPARγ modulator (SPPARM) with diverse effects in different cell types [23–25]. Given that PPARγ has also been shown to play a role in prostate development and maintenance [26], and PPARγ agonists such as troglitazone have been demonstrated to inhibit AR expression and PCa cell growth *in vitro* and *in vivo* [27–29], we reasoned that PPARγ modulation may be an unanticipated activity of EPI-001 in PCa cells. Indeed, troglitazone or EPI-001 caused inhibition of AR transcriptional activity in promoter reporter assays in LNCaP cells (Figure 4A) at doses that correlated with inhibition of AR protein expression (Figure 4B). Furthermore, treatment of LNCaP cells with troglitazone or EPI-001 resulted in dose-dependent reduction of AR protein levels as well as induction of p21 and p27 (Figure 4B). Troglitazone inhibited AR expression at lower doses than observed in prior studies [28, 30], which may be due to the absence of serum in the cell culture medium during drug treatment in our study. Finally, troglitazone treatment also inhibited the activity of AR^Gal4^, as well as the Gal4-tethered AR NTD, TAU1, and TAU5 (Figure 4C), analogous to the effect of EPI-001 in these models (Figures 1D and 1F).

**Figure 5:**
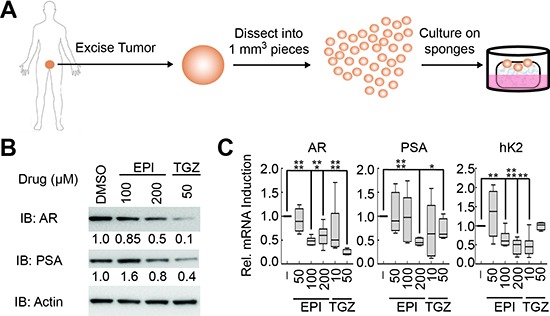
EPI-001 and troglitazone inhibit AR expression and activity in clinical prostate cancer tissues **(A)** Schematic of explant model for culturing fresh prostate cancer tissue. (*B* and *C*) PCa tissue explants were treated with EPI-001 or troglitazone (TGZ) as indicated for 48 h, then subjected to **(B)** western blot or **(C)** qRT-PCR. Box plots represent mean and range of two replicates from three patients each per treatment condition (*n* = 6). *P < 0.05; **P < 0.01, ***P < 0.001, ****P < 0.0001.

To expand these observations to clinical disease, we treated fresh human PCa tissue maintained as explants [31–33] with troglitazone and EPI-001 (Figure 5A). The doses of EPI-001 and troglitazone used in this model were increased 2- to 4-fold relative to *in vitro* experiments to reflect the higher doses of drug that have been used for *in vivo* [21] or *ex vivo* [34] experimentation. Importantly, both EPI-001 and troglitazone effected decreases in AR protein, AR mRNA, and AR target gene expression in PCa explants (Figure 5B and 5C).

### EPI-001 is a selective modulator of PPARγ in PCa cells

**Figure 6:**
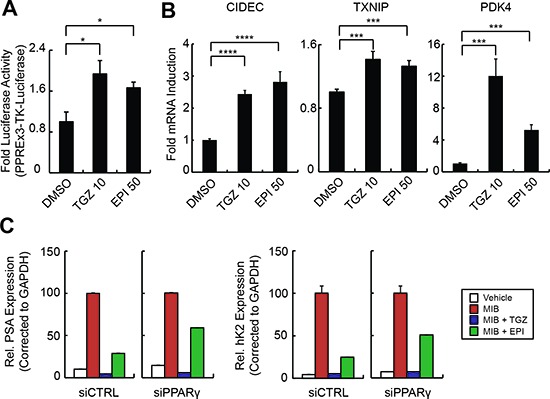
EPI-001 is a selective PPAR-γ modulator **(A)** LNCaP cells were transfected with PPREx3-TK-Luciferase, treated as indicated for 8 h, and subjected to luciferase assay. **(B)** PC-3 cells were treated with EPI-001 overnight and RNA was isolated for analysis of PPARγ target gene expression by qRT-PCR. **(C)** LNCaP cells were transfected with siRNAs targeting AR or PPARγ and treated with mibolerone (Mib), troglitazone (TGZ) or EPI-001 as indicated. Expression of AR target genes was analyzed by qRT-PCR. For *A* and *B*, bars represent mean +/− standard error (*n* = 6 from two independent triplicate experiments). For *C*, Bars represent mean +/− standard deviation (*n* = 2 from a duplicate experiment representative of 3 biological replicates). *P < 0.05; **P < 0.01, ***P < 0.001.

We next tested for SPPARM activity of EPI-001 in PCa cells. Similar to troglitazone, EPI-001 activated a PPARγ-response element (PPRE)-regulated luciferase reporter in LNCaP cells (Figure 6A). This SPPARM activity was AR-independent, as troglitazone and EPI-001 both induced mRNA expression of the PPARγ targets CIDEC [35], TXNIP [30], and PDK4 [26] in the AR-null PC-3 cell line [27] (Figure 6B). However, in 3T3-L1 cells that had been differentiated to PPARγ-positive adipocytes, EPI-001 repressed expression of classical PPARγ target genes aP2 and LPL (Supplementary Figure S12A) and inhibited lipid droplet formation (Supplementary Figure S12B). These effects are consistent with previous reports of BADGE-mediated repression of PPARγ activity in 3T3-L1 adipocytes at micromolar concentrations [23]. Taken together, these cell type-specific PPARγ agonist/antagonist effects support a SPPARM function for EPI-001, with thiazolidinedione-like effects on PPARγ activity in PCa cells.

To investigate the relationship between EPI-001-mediated PPARγ activation and AR inhibition, we knocked down PPARγ with siRNA in LNCaP cells. Despite effective silencing of PPARγ expression, both troglitazone and EPI-001 maintained robust inhibition of AR protein expression (Supplementary Figure S13A). This finding is consistent with a previous study showing that troglitazone-mediated inhibition of AR expression is due to PPARγ-independent degradation of the transcription factor Sp1 [28]. However, EPI-001 had no effect on Sp1 levels (Supplementary Figure 13B). Conversely, siRNA-mediated knock down of PPARγ partially rescued the inhibition of AR transcriptional activity effected by EPI-001, but not troglitazone (Figure 6C), demonstrating that PPARγ participates in EPI-001-mediated inhibition of AR transcriptional activity, but not inhibition of AR expression.

### EPI-001 forms covalent adducts with thiols *in vitro*

**Figure 7:**
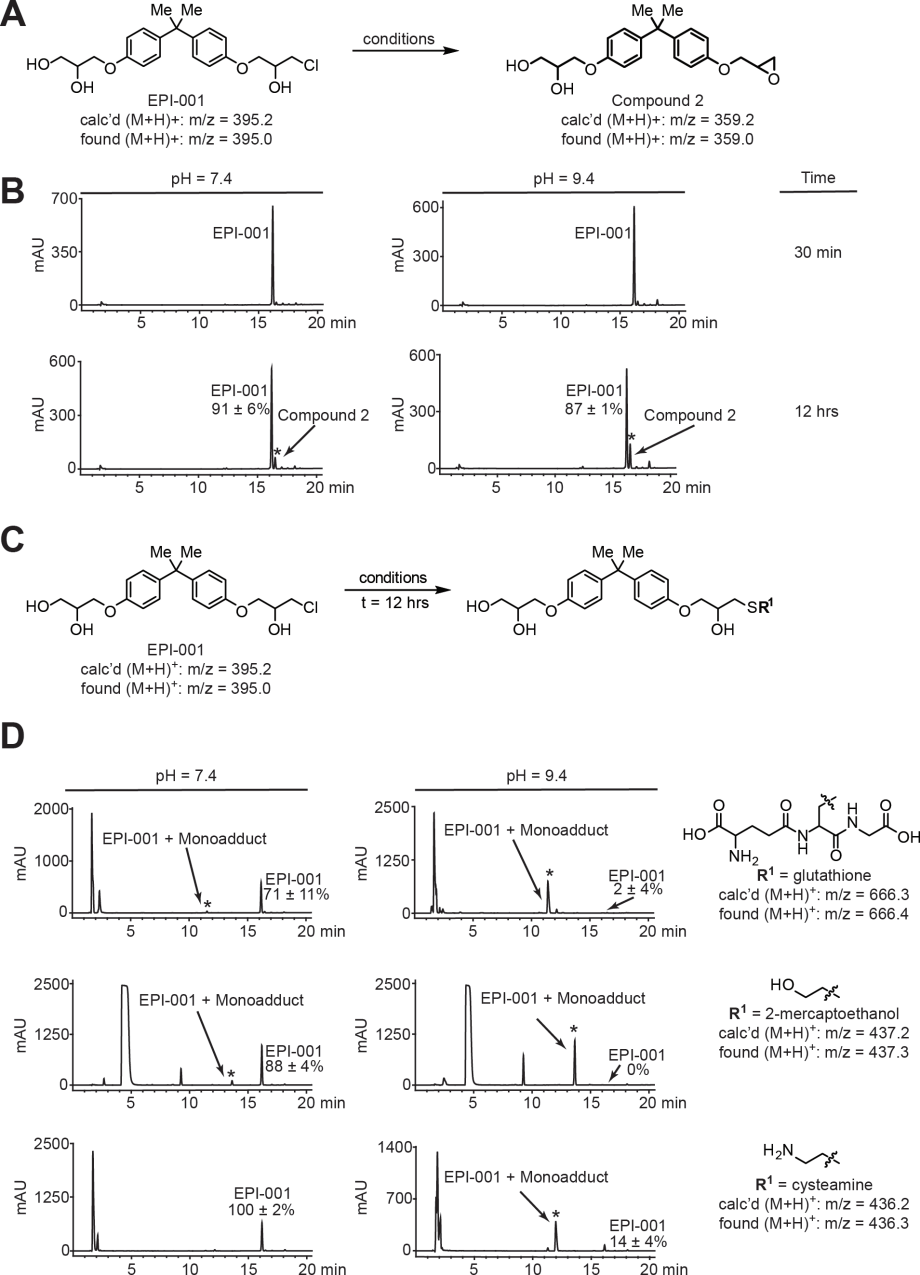
EPI-001 converts in solution to a reactive epoxide and forms covalent adducts with thiols **(A)** Scheme for conversion of EPI-001 to compound 2. EPI-001 was shaken at 37°C in PBS/DMSO at pH 2.4, 7.4, and 9.4. **(B)** HPLC chromatograms for conversion of EPI-001 to compound. Reaction mixtures were analyzed by LC-MS to confirm the presence of epoxide; *m/z* [M+H]^+^ 359.2 (calc'd); 359.0 (found). **(C)** Scheme for covalent modification of EPI-001 by reactive thiols. Solutions of EPI-001 and thiols in PBS/DMSO at pH 2.4, 7.4, and 9.4, respectively, were shaken at 37°C. **(D)** HPLC chromatograms for covalent adduct formation between EPI-001 and thiols (*t* = 12 h). New peaks that arose during the course of the reaction and are distinct from background signals (Supplementary Figure 20A) are denoted with an asterisk. Percent remaining was calculated by dividing the amount of measured EPI-001 remaining at *t* = 12 h by the amount remaining at t ~ 30 min and multiplying by 100%. Experiments were performed in triplicate and values shown are mean +/− standard deviation. EPI-001:thiol adducts were characterized by LC-MS.

Because SPPARM activity did not fully account for the multi-level anti-AR effects of EPI-001, we considered the fact that Bisphenol A (BPA) and BADGE are endocrine disruptors used in the production of polycarbonate plastics and epoxy resins [36, 37]. The epoxide rings in BADGE and related compounds readily undergo hydrolysis and hydrochlorination reactions with substrates in aqueous solution [38], resulting in hydroxylated and halogenated derivatives, of which EPI-001 (BADGE.HCl.H_2_O) is one example [39]. Chlorohydrin moieties also have the potential to spontaneously interconvert to epoxides in aqueous solution [40]. Therefore, we used HPLC to interrogate whether EPI-001 can convert to a BADGE-like mono-epoxide in solution (Compound 2, Supplementary Figure S2). Indeed, the epoxide was observed after 12h incubation at neutral and basic pH (Figure 7A and 7B), but not under acidic conditions (Supplementary Figure S14A & S14B). The identity of compound 2 was confirmed by co-injection with an authentic standard and LC-MS analysis (Supplementary Figure S15). BADGE has been shown to react with nucleophilic side chains of food proteins in plastic-lined cans [41], which is the same reaction proposed for the specific AR-binding mechanism of EPI-001 [21]. In a previous study, non-specific reactivity of EPI-001 with nucleophilic thiols was not observed [21]. However, given our observation that EPI-001 spontaneously converts to the epoxide at neutral and basic pH, and that BADGE is reactive to nucleophiles, we queried reactivity of EPI-001 with the nucleophilic thiols glutathione, 2-mercaptoethanol, and cysteamine at various pH conditions (Figure 7C). No EPI-001:thiol adducts were formed under acidic conditions (Supplementary Figure S16A & S16B, and Supplementary Figure S17). However, reaction of EPI-001 with glutathione resulted in a trace amount of thiol adduct formation at pH 7.4, and nearly complete conversion to the glutathione adduct at pH 9.4 after 12 hours (Figure 7D). Similarly, 2-mercaptoethanol displayed limited adduct formation at neutral pH, but underwent complete conversion to the EPI-001:thiol adduct at basic pH (Figure 7D). Finally, EPI-001 did not react with cysteamine at pH 7.4, but displayed nearly complete adduct formation at pH 9.4 (Figure 7D). All EPI-001-thiol adducts were confirmed by mass spectrometry (Supplementary Figure S18A–S18C). Additionally, the monoepoxide, compound 2, formed adducts with all thiols examined and displayed an enhanced reactivity profile overall (Supplementary Figure S19A–S19C). Collectively, these data indicate that EPI-001 spontaneously converts to the more reactive epoxide in solution at neutral and basic pH. Furthermore, EPI-001 extensively alkylates thiols under basic conditions with appreciable amounts of EPI-001:thiol adducts observed at neutral (7.4) pH. Our results suggest that EPI-001 is a reactive electrophile which may display some selectivity in modulation of proteins by virtue of local pH influence.

## DISCUSSION

In this study, we describe unanticipated multi-level effects of EPI-001 on the AR and PPARγ pathways, leading to inhibition of cell growth. In previous reports, EPI-001 was shown to bind specifically to the AR NTD through a nucleophilic substitution reaction with the EPI-001 chlorohydrin group [21], thereby inhibiting AR activity via occlusion of an unidentified CBP binding domain [20]. We were unable to nominate a discrete AR NTD motif that could account for a specific mechanism of EPI-001-mediated AR transcriptional repression in this study. Conversely, we found that EPI-001 inhibited synthesis of AR in PCa cell lines and clinical tissues at doses that corresponded with the inhibition of AR target genes and PCa cell growth. The LNCaP cell line was an exception to this general dose relationship between AR expression inhibition and cell growth inhibition, displaying the highest sensitivity to EPI-001- and BABDHE-mediated growth inhibition. This is important, as the majority of pre-clinical data supporting the efficacy and specificity of EPI-001 for AR has been generated using the LNCaP model [20, 21]. Moreover, we found that EPI-001 inhibited the growth of AR-negative PC-3 and DU 145 cells. These data conflict with a previous report [20], but we propose that this discrepancy is due to two key differences in experimental design. First, our study incorporated longer-term (i.e. 7 day) growth assays as opposed to early timepoint (i.e. 3 day) BrdU incorporation readouts. Secondly, previous reports used 10 μM EPI-001 to treat PC-3 and DU 145, a dose which was not inhibitory to the growth of PC-3 and DU 145 in our study, but inhibitory LNCaP cells. These data highlight the cell line-specific responses to EPI-001, which supported earlier conclusions of AR specificity.

Our data indicate that PPARγ activation represents at least one AR-independent activity of EPI-001 in PCa cells. However, EPI-001 displayed PPARγ inhibitory activity in a classical 3T3-L1 adipocyte model, indicating SPPARM activity as opposed to pure agonist activity. SPPARM activity for EPI-001 is consistent with studies demonstrating that the chemically-related compound, BADGE, is a SPPARM that binds to the PPARγ LBD [23] and exhibits distinct molecular effects in PCa and 3T3-L1 cells when compared with synthetic thiazolidinedione PPARγ agonists [23–25] including troglitazone [36–38]. Furthermore, our data from thiol reactivity assays demonstrate that small molecule thiolates (e.g., glutathione, 2-mercaptoethanol, cysteamine) are readily alkylated by EPI-001 and this reactivity is attenuated at acid and neutral pH. Consequently, our data suggest that any protein bearing an accessible nucleophilic residue within a suitably basic binding pocket may be a target for covalent modification by EPI-001. This is further supported by the established reactivity of BADGE *in vitro* [41], and our data that EPI-001 is converted to an analogous epoxide (compound 2) in solution at physiological pH. Collectively, these data suggest EPI-001 and BADGE bear substantial proteome reactivity features in addition to their reported interactions with AR and PPARγ.

These new data indicate that structural changes to the core bisphenol of EPI-001 as well as the covalent warhead may be required to mitigate the AR-independent effects reported in this study and in the toxicology literature [36–38, 41, 42]. However, this task is complicated because no 3-dimensional structure has been reported for the intrinsically disordered AR NTD [43, 44], which impedes the rational design of improved inhibitors. Nevertheless, EPI-002, the (2*R*, 20*S*) isomer of racemic EPI-001, has been shown to display stronger AR interactions and reduced toxicity in mice [21], indicating this direction may be feasible. Our findings that EPI-001-mediated inhibition of AR activity is associated with inhibition of AR expression and activation of PPARγ in PCa, coupled with the finding that EPI-001 can capture nucleophilic thiols, will be important for ongoing pre-clinical development of EPI-001 and other anti-AR compounds that target functional domains independent of the AR LBD.

## MATERIALS AND METHODS

### Cell culture and growth assays

LNCaP, C4-2, DU 145, VCaP, 22Rv1, 293T, and PC-3 cell lines were obtained from ATCC. The ATCC validates the authenticity of these cell lines via short tandem repeat (STR) analysis. CWR-R1 prostate cancer cells were the generous gift of Dr. Elizabeth Wilson (University of North Carolina at Chapel Hill, Chapel Hill, NC). CWR-R1 cells were authenticated by sequence-based validation of two characteristic AR mutations: a H874Y mutation in the LBD, and a 50 kb deletion in AR intron 1 [45]. VCaP and 293T were maintained in Dulbecco's Modified Eagle's Medium (DMEM) with 10% FBS. All other cell lines were maintained in RPMI 1640 medium with 10% FBS, and all cell lines were maintained in 100 Units/mL Penicillin + 0.1 mg/mL Streptomycin. Cells were cultured in a 37°C incubator with 5% CO_2_ for no longer than 15 passages after resuscitation of frozen stocks. Cell growth was assessed by crystal violet staining as previously described [46].

### Reagents

Dihydrotestosterone (DHT), mibolerone (MIB), and BABDHE (Bisphenol A bis [2,3-dihydroxypropyl] ether) were purchased from Sigma. Enzalutamide (ENZ) was purchased from Selleck Chemicals. Troglitazone was purchased from Cayman Chemical. EPI-001 (Bisphenol A [3-chloro-2-hydroxypropyl] [2,3-dihydroxypropyl] ether) was synthesized (See Supplementary Methods) or purchased from commercial sources (Santa Cruz Biotechnology or Sigma-Aldrich). EPI-001 was analyzed for purity via HPLC and NMR (Supplementary Methods, Supplementary Figures S1 and S2). EPI-001 dissolved in DMSO was used for all experiments (Supplementary Methods, Supplementary Figure S3). All other drugs were suspended in DMSO with the exception of DHT, MIB, and ENZ, which were prepared in absolute ethanol. Final DMSO or ethanol concentrations did not exceed 0.1% (v/v) in culture medium.

### Plasmids

Plasmids encoding human AR (p5HBhAR-A), AR^Gal4^, NTD-Gal4, AR^Gal4^ΔTAU1, AR^Gal4^ΔTAU5, sPSA-Luciferase (also referred to as PSAenh(ARE)-LUC), and sPSA^Gal4^-Luciferase (also referred to as PSAenh(GAL4)-LUC) have been described [22]. SV40-Renilla, CMV-Renilla, and pG5-Luciferase were purchased from Promega. PPREx3-TK-Luciferase has been described [47], and was obtained from Addgene. The Gal4 DBD expression plasmid (pM) was purchased from Clontech. Gal4-TAU1 (AR a.a. 101–360) and Gal4-TAU5 (AR a.a. 361–490) were constructed as described in the Supplementary Methods and Supplementary Table 1.

### Cell transfection

LNCaP cells were transfected via single-pulse electroporation as previously described [48]. C4-2 cells were transfected with Superfect reagent (Qiagen) according to manufacturer specifications. 293T cells were transfected with Lipofectamine 2000 (Life Technologies) according to manufacturer specifications. Treatment of transfected cells with androgen and/or drug was performed for 8 hours or overnight in serum-free medium as indicated.

### Dual luciferase assays

Transfected cells were lysed in 1X Passive Lysis Buffer (Promega) and subjected to dual luciferase assays using a Dual Luciferase Assay Kit (Promega) as previously described [48].

### Western blot

Western blotting with antibodies listed in Supplementary Table 2 was performed as previously described [49].

### Nascent RNA labeling and isolation

Nascent transcripts were isolated using the Click-iT Nascent RNA Capture Kit (Life Technologies) according to manufacturer specifications. First-strand cDNA synthesis was performed using the SuperScript VILO cDNA synthesis kit (Life Technologies) according to manufacturer specifications and quantified via qRT-PCR. Detailed information regarding reagent concentrations and time of treatment can be found in the Supplementary Methods.

### Quantitative RT-PCR

RNA isolation and quantitative RT-PCR analysis were performed as described [49] using primers listed in Supplementary Table 3.

### Prostate cancer explants

Patient tissues were obtained from the University of Texas Southwestern Medical Center tissue core under UTSW IRB STU 112013–056 and explant studies were performed as previously described [31, 32]. Detailed information on tissue treatment, dosing, timing, and processing can be found in the Supplementary Methods.

### pH stability and thiol reactivity studies

Solutions of reduced l-glutathione, 2-mercaptoethanol, and cysteamine in phosphate buffered saline were adjusted to the desired pH (2.4, 7.4, or 9.4). EPI-001 or monoepoxide control (Compound 2, Supplementary Figure S2) were added to thiol solutions and aliquots of reactions were analyzed by reverse phase HPLC and LC-MS at the indicated time points. To quantify the amount of parent compound remaining, the area under the curve (AUC) of the parent compound was divided by the AUC of an internal standard. Further information regarding thiol reactivity, HPLC, and LC-MS conditions are included in the Supplementary Methods.

### Data analysis and statistics

All statistical comparisons were made using the two-tailed Student's *t*-Test with a P value of 0.05 or less considered significant.

## SUPPLEMENTARY METHODS, FIGURES AND TABLES



## References

[1] Siegel R, Ma J, Zou Z, Jemal A (2014). Cancer statistics, 2014. CA: a cancer journal for clinicians.

[2] Barfeld S, Itkonen H, Urbanucci A, Mills I (2014). Androgen-regulated metabolism and biosynthises in prostate cancer. Endocrine-related cancer.

[3] Culig Z, Santer FR (2014). Androgen receptor signaling in prostate cancer. Cancer metastasis reviews.

[4] Helsen C, Van den Broeck T, Voet A, Prekovic S, Van Poppel H, Joniau S, Claessens F (2014). Androgen receptor antagonists for prostate cancer therapy. Endocrine-related cancer.

[5] Petrylak DP (2013). Current state of castration-resistant prostate cancer. The American journal of managed care.

[6] Scher HI, Fizazi K, Saad F, Taplin ME, Sternberg CN, Miller K, de Wit R, Mulders P, Chi KN, Shore ND, Armstrong AJ, Flaig TW, Flechon A (2012). Increased survival with enzalutamide in prostate cancer after chemotherapy. N Engl J Med.

[7] Sternberg CN, de Bono JS, Chi KN, Fizazi K, Mulders P, Cerbone L, Hirmand M, Forer D, Scher HI (2014). Improved outcomes in elderly patients with metastatic castration-resistant prostate cancer treated with the androgen receptor inhibitor enzalutamide: results from the phase III AFFIRM trial. Ann Oncol.

[8] Beer TM, Armstrong AJ, Rathkopf DE, Loriot Y, Sternberg CN, Higano CS, Iversen P, Bhattacharya S, Carles J, Chowdhury S, Davis ID, de Bono JS, Evans CP (2014). Enzalutamide in Metastatic Prostate Cancer before Chemotherapy. N Engl J Med.

[9] Ryan CJ, Smith MR, de Bono JS, Molina A, Logothetis CJ, de Souza P, Fizazi K, Mainwaring P, Piulats JM, Ng S, Carles J, Mulders PF, Basch E (2013). Abiraterone in metastatic prostate cancer without previous chemotherapy. N Engl J Med.

[10] Arora VK, Schenkein E, Murali R, Subudhi SK, Wongvipat J, Balbas MD, Shah N, Cai L, Efstathiou E, Logothetis C, Zheng D, Sawyers CL (2013). Glucocorticoid receptor confers resistance to antiandrogens by bypassing androgen receptor blockade. Cell.

[11] Chang KH, Li R, Kuri B, Lotan Y, Roehrborn CG, Liu J, Vessella R, Nelson PS, Kapur P, Guo X, Mirzaei H, Auchus RJ, Sharifi N (2013). A gain-of-function mutation in DHT synthesis in castration-resistant prostate cancer. Cell.

[12] Li Y, Chan SC, Brand LJ, Hwang TH, Silverstein KA, Dehm SM (2013). Androgen receptor splice variants mediate enzalutamide resistance in castration-resistant prostate cancer cell lines. Cancer research.

[13] Mostaghel EA, Marck BT, Plymate SR, Vessella RL, Balk S, Matsumoto AM, Nelson PS, Montgomery RB (2011). Resistance to CYP17A1 inhibition with abiraterone in castration-resistant prostate cancer: induction of steroidogenesis and androgen receptor splice variants. Clinical cancer research:an official journal of the American Association for Cancer Research.

[14] Nyquist MD, Li Y, Hwang TH, Manlove LS, Vessella RL, Silverstein KA, Voytas DF, Dehm SM (2013). TALEN-engineered AR gene rearrangements reveal endocrine uncoupling of androgen receptor in prostate cancer. Proc Natl Acad Sci U S A.

[15] Mateo J, Smith A, Ong M, de Bono JS (2014). Novel drugs targeting the androgen receptor pathway in prostate cancer. Cancer metastasis reviews.

[16] Dehm SM, Tindall DJ (2007). Androgen receptor structural and functional elements: role and regulation in prostate cancer. Mol Endocrinol.

[17] Claessens F, Denayer S, Van Tilborgh N, Kerkhofs S, Helsen C, Haelens A (2008). Diverse roles of androgen receptor (AR) domains in AR-mediated signaling. Nuclear receptor signaling.

[18] Brand LJ, Dehm SM (2013). Androgen receptor gene rearrangements: new perspectives on prostate cancer progression. Current drug targets.

[19] Nyquist MD, Dehm SM (2013). Interplay between genomic alterations and androgen receptor signaling during prostate cancer development and progression. Hormones & cancer.

[20] Andersen RJ, Mawji NR, Wang J, Wang G, Haile S, Myung JK, Watt K, Tam T, Yang YC, Banuelos CA, Williams DE, McEwan IJ, Wang Y (2010). Regression of castrate-recurrent prostate cancer by a small-molecule inhibitor of the amino-terminus domain of the androgen receptor. Cancer cell.

[21] Myung JK, Banuelos CA, Fernandez JG, Mawji NR, Wang J, Tien AH, Yang YC, Tavakoli I, Haile S, Watt K, McEwan IJ, Plymate S, Andersen RJ (2013). An androgen receptor N-terminal domain antagonist for treating prostate cancer. The Journal of clinical investigation.

[22] Dehm SM, Tindall DJ (2006). Ligand-independent androgen receptor activity is activation function-2-independent and resistant to antiandrogens in androgen refractory prostate cancer cells. The Journal of biological chemistry.

[23] Wright HM, Clish CB, Mikami T, Hauser S, Yanagi K, Hiramatsu R, Serhan CN, Spiegelman BM (2000). A synthetic antagonist for the peroxisome proliferator-activated receptor gamma inhibits adipocyte differentiation. The Journal of biological chemistry.

[24] Nakamuta M, Enjoji M, Uchimura K, Ohta S, Sugimoto R, Kotoh K, Kato M, Irie T, Muta T, Nawata H (2002). Bisphenol a diglycidyl ether (BADGE) suppresses tumor necrosis factor-alpha production as a PPARgamma agonist in the murine macrophage-like cell line, RAW 264.7. Cell biology international.

[25] Dworzanski T, Celinski K, Korolczuk A, Slomka M, Radej S, Czechowska G, Madro A, Cichoz-Lach H (2010). Influence of the peroxisome proliferator-activated receptor gamma (PPAR-gamma) agonist, rosiglitazone and antagonist, biphenol-A-diglicydyl ether (BADGE) on the course of inflammation in the experimental model of colitis in rats. Journal of physiology and pharmacology:an official journal of the Polish Physiological Society.

[26] Strand DW, Jiang M, Murphy TA, Yi Y, Konvinse KC, Franco OE, Wang Y, Young JD, Hayward SW (2012). PPARgamma isoforms differentially regulate metabolic networks to mediate mouse prostatic epithelial differentiation. Cell death & disease.

[27] Kubota T, Koshizuka K, Williamson EA, Asou H, Said JW, Holden S, Miyoshi I, Koeffler HP (1998). Ligand for peroxisome proliferator-activated receptor gamma (troglitazone) has potent antitumor effect against human prostate cancer both *in vitro* and *in vivo*. Cancer research.

[28] Yang CC, Wang YC, Wei S, Lin LF, Chen CS, Lee CC, Lin CC (2007). Peroxisome proliferator-activated receptor gamma-independent suppression of androgen receptor expression by troglitazone mechanism and pharmacologic exploitation. Cancer research.

[29] Sikka S, Chen L, Sethi G, Kumar AP (2012). Targeting PPARgamma Signaling Cascade for the Prevention and Treatment of Prostate Cancer. PPAR research.

[30] Hisatake JI, Ikezoe T, Carey M, Holden S, Tomoyasu S, Koeffler HP (2000). Down-Regulation of prostate-specific antigen expression by ligands for peroxisome proliferator-activated receptor gamma in human prostate cancer. Cancer research.

[31] Centenera MM, Gillis JL, Hanson AR, Jindal S, Taylor RA, Risbridger GP, Sutherland PD, Scher HI, Raj GV, Knudsen KE, Yeadon T, Tilley WD, Butler LM (2012). Evidence for efficacy of new Hsp90 inhibitors revealed by *ex vivo* culture of human prostate tumors. Clinical cancer research:an official journal of the American Association for Cancer Research.

[32] Centenera MM, Raj GV, Knudsen KE, Tilley WD, Butler LM (2013). *Ex vivo* culture of human prostate tissue and drug development. Nature reviews Urology.

[33] Schiewer MJ, Goodwin JF, Han S, Brenner JC, Augello MA, Dean JL, Liu F, Planck JL, Ravindranathan P, Chinnaiyan AM, McCue P, Gomella LG, Raj GV (2012). Dual roles of PARP-1 promote cancer growth and progression. Cancer discovery.

[34] Wang S, Kollipara RK, Srivastava N, Li R, Ravindranathan P, Hernandez E, Freeman E, Humphries CG, Kapur P, Lotan Y, Fazli L, Gleave ME, Plymate SR (2014). Ablation of the oncogenic transcription factor ERG by deubiquitinase inhibition in prostate cancer. Proc Natl Acad Sci U S A.

[35] Puri V, Ranjit S, Konda S, Nicoloro SM, Straubhaar J, Chawla A, Chouinard M, Lin C, Burkart A, Corvera S, Perugini RA, Czech MP (2008). Cidea is associated with lipid droplets and insulin sensitivity in humans. Proc Natl Acad Sci U S A.

[36] Simal-Gandara J, Paz-Abuin S, Ahrne L (1998). A critical review of the quality and safety of BADGE-based epoxy coatings for cans: implications for legislation on epoxy coatings for food contact. Crit Rev Food Sci Nutr.

[37] Delfosse V, Grimaldi M, le Maire A, Bourguet W, Balaguer P (2014). Nuclear receptor profiling of bisphenol-A and its halogenated analogues. Vitam Horm.

[38] Hammarling L, Gustavsson H, Svensson K, Oskarsson A (2000). Migration of bisphenol-A diglycidyl ether (BADGE) and its reaction products in canned foods. Food Addit Contam.

[39] Lintschinger J, Rauter W (2000). Simultaneous determination of bisphenol A-diglycidyl ether, bisphenol F-diglycidyl ether and their hydrolysis and chlorohydroxy derivatives in canned foods. Eur Food Res Technol.

[40] Agatsuma T, Ogawa H, Akasaka K, Asai A, Yamashita Y, Mizukami T, Akinaga S, Saitoh Y (2002). Halohydrin and oxime derivatives of radicicol: Synthesis and antitumor activities. Bioorgan Med Chem.

[41] Petersen H, Biereichel A, Burseg K, Simat TJ, Steinhart H (2008). Bisphenol A diglycidyl ether (BADGE) migrating from packaging material ‘disappears’ in food: reaction with food components. Food Addit Contam Part A Chem Anal Control Expo Risk Assess.

[42] Bakhiya N, Abraham K, Gurtler R, Appel KE, Lampen A (2011). Toxicological assessment of 3-chloropropane-1,2-diol and glycidol fatty acid esters in food. Mol Nutr Food Res.

[43] Lavery DN, McEwan IJ (2008). Structural characterization of the native NH2-terminal transactivation domain of the human androgen receptor: a collapsed disordered conformation underlies structural plasticity and protein-induced folding. Biochemistry.

[44] McEwan IJ (2012). Intrinsic disorder in the androgen receptor: identification, characterisation and drugability. Molecular bioSystems.

[45] Li Y, Hwang TH, Oseth LA, Hauge A, Vessella RL, Schmechel SC, Hirsch B, Beckman KB, Silverstein KA, Dehm SM (2012). AR intragenic deletions linked to androgen receptor splice variant expression and activity in models of prostate cancer progression. Oncogene.

[46] Li Y, Alsagabi M, Fan D, Bova GS, Tewfik AH, Dehm SM (2011). Intragenic rearrangement and altered RNA splicing of the androgen receptor in a cell-based model of prostate cancer progression. Cancer research.

[47] Kim JB, Wright HM, Wright M, Spiegelman BM (1998). ADD1/SREBP1 activates PPARgamma through the production of endogenous ligand. Proc Natl Acad Sci U S A.

[48] Dehm SM, Schmidt LJ, Heemers HV, Vessella RL, Tindall DJ (2008). Splicing of a novel androgen receptor exon generates a constitutively active androgen receptor that mediates prostate cancer therapy resistance. Cancer research.

[49] Li Y, Alsagabi M, Fan D, Bova GS, Tewfik AH, Dehm SM (2011). Intragenic rearrangement and altered RNA splicing of the androgen receptor in a cell-based model of prostate cancer progression. Cancer research.

