# Supplementary material for: EPI-001 is a selective peroxisome proliferator-activated receptor-gamma modulator with inhibitory effects on androgen receptor expression and activity in prostate cancer
Source: Oncotarget. 2015 Feb 11;6(6):3811–24. doi: 10.18632/oncotarget.2924 (PMC4414155; doi:10.18632/oncotarget.2924)
Supplement: Supplementary file 1 [file oncotarget-06-3811-s001.pdf]

# SUPPLEMENTARY METHODS

## Synthesis of EPI-001

### General

Chemical reagents were typically purchased from Sigma-Aldrich and used without additional purification unless noted. Bulk solvents were from Fisher Scientific. N,N-dimethylformamide (DMF) was rendered anhydrous by passing through the resin column of a solvent purification system (MBraun). Reactions were performed under an atmosphere of dry $N_2$ unless noted. Silica gel chromatography was preformed on a Teledyne-Isco Combiflash Rf-200 instrument utilizing Redisep R*f* Gold High Performance silica gel columns (Teledyne-Isco). Analytical HPLC analysis was performed on an Agilent 1200 series instrument equipped with a diode array detector and a Zorbax SB-C18 column (4.6 × 150 mm, 3.5 µm, Agilent Technologies). The method started with a 10% $CH_3CN$ (with 0.1% trifluoroacetic acid (TFA)) in $H_2O$ (0.1% TFA), the 10% $CH_3CN$ (with 0.1% TFA) was increased to 15% over 2 minutes, increased to 20% over 3 more minutes and then increased to 95% $CH_3CN$ (with 0.1% TFA) over 25 minutes. Nuclear magnetic resonance (NMR) spectroscopy employed a Bruker Ascend instrument operating at 500 MHz (for $^1$H) and 125 MHz (for $^{13}$C) at ambient temperature. Chemical shifts are reported in parts per million and normalized to internal solvent peaks or tetramethylsilane. Mass spectrometry was recorded in positive-ion mode on an Agilent MSD SL Ion Trap.

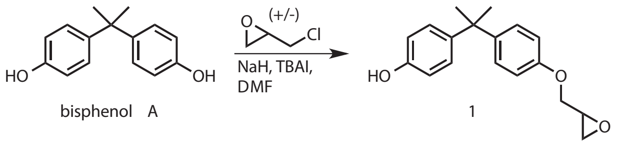


(4-hydroxyphenyl)(4-(oxiran-2-ylmethoxy)phenyl) methanone (1). This compound was prepared by modification to the previously reported procedure [1]. To a stirred solution of NaH (60% dispersion in oil, 1.453 g, 36.331 mmol) in anhydrous DMF (150 mL) at 0°C was added bisphenol A (4.000 g, 16.514 mmol). The reaction was stirred for 30 min, then tetrabutylammonium iodide (1.220 g, 3.303 mmol) and epichlorohydrin (3.24 mL, 41.3 mmol) was added and the reaction was allowed to warm up to room temperature (rt) over 12 h. The reaction was quenched with water and extracted with ethyl acetate (3 × 50 mL). The organic layer was washed with water (2 × 30 mL), dried over $Na_2SO_4$, and concentrated *in vacuo*. The crude mixture was purified by silica gel chromatography (gradient $CH_2Cl_2$ to 10% EtOAc in $CH_2Cl_2$) to afford 1 (0.580 g, 12% yield) as a clear foam. $^1$H NMR (DMSO-$d_6$): δ 9.15 (s, 1H), 7.09 (d, J = 8.5 Hz, 2 H), 6.98 (d, J = 8.5 Hz, 2 H), 6.83 (d, J = 8.5 Hz, 2 H), 6.64 (d, J = 8.5 Hz, 2 H), 4.26 (dd, J = 11.5, 2.5 Hz, 1 H), 3.78 (dd, J = 11.0, 6.5 Hz, 1 H), 3.29 (br s, 1 H), 2.82 (t, J = 4.5 Hz, 1 H), 2.69 (dd, J = 5.0, 2.5 Hz, 1 H), 1.55 (s, 6 H). $^{13}$C NMR (DMSO-$d_6$): δ 155.9, 154.9, 143.2, 140.7, 127.4 (2), 127.3 (2), 114.6 (2), 113.8 (2), 68.8, 49.7, 43.8, 41.0, 30.8 (2). MS (m/z) calc'd for $C_{18}H_{20}O_3$ 284.1, found 285.1 [M + H]+.

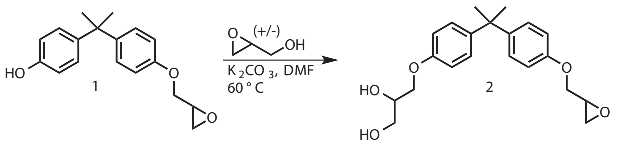


(4-(2,3-dihydroxypropoxy)phenyl)(4-(oxiran-2-ylmethoxy)phenyl)methanone (2). This compound was prepared as previously described [1]. To a stirred solution of 1 (0.493 g, 1.735 mmol) in anhydrous DMF (15 mL) at rt was added $K_2CO_3$ (0.480 g, 3.469 mmol) and glycidol (0.35 mL, 5.20 mmol). The reaction was stirred at 60°C for 12 h. The reaction was cooled to rt, quenched with water and extracted with ethyl acetate (3 × 15 mL). The reaction mixture was washed with water (2 × 10 mL), dried over $Na_2SO_4$, and concentrated *in vacuo*. The crude mixture was purified by silica gel chromatography (gradient 10% EtOAc in $CH_2Cl_2$ to 50% EtOAc in $CH_2Cl_2$) to afford 2 (0.258 g, 41% yield) as a clear foam. $^1$H NMR (DMSO-$d_6$): δ 7.09 (dd, J = 9.0, 7.0 Hz, 4 H), 6.83 (dd, J = 14.0, 8.5 Hz, 4 H), 4.89 (d, J = 5.0 Hz, 1 H), 4.62 (t, J = 5.5 Hz, 1 H), 4.26 (dd, J = 11.5, 2.5 Hz, 1 H), 3.94 (dd, J = 9.5, 4.0 Hz, 1 H), 3.82–3.74 (m, 3 H), 3.42 (t, J = 5.5 Hz, 2 H), 3.30 (br s, 1 H), 2.82 (t, J = 4.5 Hz, 1 H), 2.69 (dd, J = 5.0, 2.5 Hz, 1 H), 1.57 (s, 6 H). $^{13}$C NMR (DMSO-$d_6$): δ 156.5, 156.0, 143.0, 142.4, 127.4 (2), 127.3 (2), 113.9 (2), 113.8 (2), 69.9, 69.4, 68.8, 62.7, 49.7, 43.7, 41.1, 30.7 (2). MS (m/z) calc'd for $C_{21}H_{26}O_5$ 358.2, found 381.2 [M + Na]+.

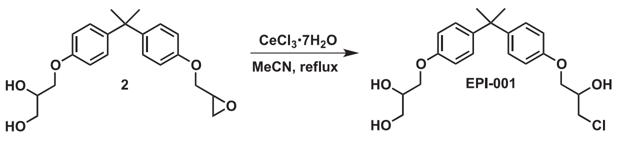


### EPI-001

[1–4] This compound was prepared by modification to the previously reported procedure. [1] To a stirred solution of 2 (0.164 g, 0.458 mmol) in $CH_3CN$ (10 mL) was added $CeCl_3 \times 7H_2O$ (0.427, 1.146 mmol) and mixture was heated to reflux for 12 h. The reaction was cooled to rt, excess $CH_2Cl_2$ was added to the white paste and

the cerium salts were removed by filtration. The filtrate was concentrated *in vacuo*. The resulting crude material was purified by silica gel chromatography (gradient 50% EtOAc in $CH_2Cl_2$ to 100% EtOAc) to afford EPI-001 (0.117 g, 64% yield) as a clear foam. $^1$H NMR (DMSO-$d_6$): δ 7.09 (dd, J = 8.5, 5.0 Hz, 4 H), 6.82 (t, J = 9.5 Hz, 4 H), 5.52 (d, J = 5.5 Hz, 1 H), 4.90 (d, J = 5.0 Hz, 1 H), 4.63 (t, J = 5.5 Hz, 1 H), 4.02–3.99 (m, 1 H), 3.95–3.92 (m, 3 H), 3.82–3.72 (m, 3 H), 3.65 (dd, J = 11.0, 5.0 Hz, 1 H), 3.42 (t, J = 6.0 Hz, 2 H), 1.57 (s, 6 H) $^{13}$C NMR (DMSO-$d_6$): δ 156.5, 156.1, 142.9, 142.4, 127.4 (2), 127.3 (2), 113.9 (2), 113.8 (2), 69.9, 69.4, 68.8, 68.6, 62.7, 46.8, 41.1, 30.7 (2). MS (m/z) calc'd for $C_{21}H_{27}ClO_5$ 394.2, found 417.2 [M + Na]+.

### Cell culture

T47D breast cancer cells were the generous gift of Dr. Carol Lange (University of Minnesota, Minneapolis, MN). Cells were maintained in Minimal Essential Medium (MEM) supplemented with 5% Fetal Bovine Serum (FBS), 5 mg/mL insulin, 2 mM L-glutamine, 100 Units/mL Penicillin, and 0.1 mg/mL Streptomycin. 3T3-L1 cells (kindly provided by Dr. David Bernlohr from the Department of Biochemistry, Molecular Biology and Biophysics, University of Minnesota) were cultured in DMEM with 10% bovine calf serum (Sigma Aldrich, Saint Louis, MO) and 100 IU/ml penicillin/streptomycin (Invitrogen, Carlsbad, CA) until confluence. Two days after confluence, cells were subjected to two-day incubation in the adipocyte differentiation cocktail containing 10% fetal bovine serum (JRH Biosciences, Inc., Lenexa, KS), 115 mg/ml methylisobutylxanthine (Sigma Aldrich, Saint Louis, MO), 1mg/ml insulin (Sigma Aldrich, Saint Louis, MO), and 390 ng/ml dexamethasone (Sigma Aldrich, Saint Louis, MO). During the following 6 days, cells were maintained in DMEM containing 10% fetal bovine serum, 100 IU/ml penicillin/streptomycin and 1mg/ml insulin until fully differentiated with robust accumulation of lipid droplets. In the treated groups, Rosiglitazone at the concentration of 1 µM or EPI at various concentrations was added to the cultures during the entire 8-day treatment period. On day 8, cells were stained with Oil-red O or harvested for RNA extraction.

### Plasmids

Gal4 tethering of AR TAU1 (amino acids 101–360) and TAU5 (amino acids 361–490) was achieved by PCR amplification of TAU1 or TAU5 from p5HBhAR-A using primers listed in Supplementary Table 1. Primers were designed to introduce in-frame recognition sites for EcoRI and BamHI, which were used to ligate the insert with EcoRI/BamHI-digested pM.

### Cell transfection

LNCaP cells were transfected with siRNA (300 pmol) or reporter/transactivator plasmids (12 ug total DNA) via single-pulse electroporation at 305V for 10 ms using an ECM 830 Square-Wave cuvette electroporator (Harvard Apparatus) with 4.0 mm gap-width cuvettes, followed by seeding in RPMI + 10% FBS. C4-2 were transfected with Superfect reagent (Qiagen) according to manufacturer specifications. Transfections were performed in RPMI + 10% CSS, and cells were incubated at 37°C for 24 hours post transfection prior to further treatment. 293T were transfected with Lipofectamine 2000 (Life Technologies) according to manufacturer specifications. Cells were transfected overnight in serum-free DMEM, then fed with fresh DMEM + 10% FBS the following morning. Treatment of transfected cells with androgen and/or drug was performed for 8 hours or overnight in serum-free medium as indicated. Transfected cells were then harvested in assay-specific lysis buffers.

### Western blot

Adherent cells were treated for 24 hours in serum-free RPMI containing drugs (1 nM DHT and/or EPI-001 at indicated doses). Cells were lysed in Laemmli buffer and equal masses of crude lysates were separated in denaturing polyacrylamide gels followed by transfer to nitrocellulose membranes (Bio-Rad). Membranes were blocked with 2.5% non-fat dry milk (w/v) in Tris-buffered saline with 1% Tween-20 (v/v) and probed with primary antibodies listed in Supplementary Table 2, followed by washing and incubation with horseradish peroxidase-conjugated secondary antibody (Santa Cruz). Chemiluminescence detection was performed using Super Signal West Pico reagent (Thermo) or WesternBright ECL (BioExpress) on HyBlot CL autoradiography film (Denville Scientific). Band intensity was assessed using NIH ImageJ software.

### Proteasome inhibition

LNCaP and C4-2 were seeded in RPMI + 10% CSS. Cells were then serum-starved overnight and co-treated with 10 µM MG-132 (Sigma) and/or 50 µM EPI-001 and harvested at the indicated time points for western blot.

### mRNA stability assay

LNCaP were seeded in RPMI + 10% CSS for 48 hours, then serum-starved overnight and co-treated with 10 µg/mL Actinomycin D (Sigma) and 50 µM EPI-001 in serum-free RPMI as indicated. Cells were harvested in guanidinium thiocyanate buffer at the indicated time points and RNA was extracted and analyzed via qRT-PCR.

## Nascent RNA labeling and isolation

Nascent transcripts were labeled with biotin and subjected to streptavidin pull-down using the Click-iT Nascent RNA Capture Kit (Life Technologies) according to manufacturer specifications. Briefly, LNCaP cells were serum-starved overnight, then treated with 50 µM EPI-001 or vehicle control. After 7 hours incubation, cells were pulsed with 5-ethynyl Uridine (5EU) for an additional hour in the presence of EPI-001 or vehicle to label nascent transcripts, then harvested in Trizol (Life Technologies). Total RNA was then subjected to a Click chemistry reaction which attached a biotin molecule to 5EU-labeled nascent transcripts. RNA was re-precipitated, then bound to streptavidin-conjugated magnetic beads and washed 10X to remove unlabeled transcripts, leaving only biotin-5EU-labeled nascent RNA attached to the beads. First-strand cDNA synthesis was performed directly on RNA:bead conjugates using the SuperScript VILO cDNA synthesis kit (Life Technologies) according to manufacturer specifications, then subjected to qRT-PCR as described below.

## Quantitative RT-PCR

LNCaP, C4-2, and PC-3 cells were seeded in RPMI 1640 with 10% CSS for 48 h to allow androgen signaling to nadir prior to drug treatment. Cells were co-treated with 50µM EPI-001 and/or 1 nM mibolerone or appropriate vehicle controls as indicated. RNA was extracted using an acid guanidinium thiocyanate-phenol-chloroform method as described [5] and reverse transcribed with a Transcriptor First-Strand cDNA synthesis kit (Roche) according to manufacturer specifications. Quantitative PCR for AR pre-mRNA and mature mRNA, PSA, hK2, TXNIP, CIDEC, LPL, FABP4 (aP2), PDK4, and either TBP, GAPDH, or Actin as housekeeping controls was performed using PerfeCTa SYBR Green Fastmix (Quanta Bioscience) according to manufacturer specifications. Primer sequences used in qPCR assays are listed in Supplementary Table 3. Fluorescence intensity was evaluated after every PCR cycle using a Bio-Rad My iQ thermal cycler set for default 2-step amplification (40 cycles). Gene expression was normalized to housekeeping controls (TBP, GAPDH, or Actin) using the formula $2^{-\Delta\Delta Ct}$, where $\Delta\Delta Ct$ = threshold cycle of amplification difference between the gene of interest and the housekeeping control.

## Prostate cancer explants

Patient tissues were obtained from the University of Texas Southwestern Medical Center tissue core under UTSW IRB STU 112013–056 and explant studies were performed as previously described [6, 7]. Briefly, cancerous prostates were excised from patients with high-risk (GG8–10) high volume (> 2 positive cores) prostate cancers via robotic laparoscopic prostatectomy. Pathology-validated cancer tissue was dissected into 1 $mm^3$ cubes, and cultured on Surgifoam oral gelatin sponges (Ethicon) for 24 hours in RPMI + 10% FBS supplemented with insulin and hydrocortisone at 10 mg/L each. Explants were then washed 3 × 1 hr in RPMI + 10% CSS supplemented +/– 1 nM Mibolerone alone or in combination with Troglitazone (10 or 50 µM) or EPI-001 (50, 100, or 200 µM) as indicated. Tissue explants were then cultured in RPMI + 10% CSS supplemented with 1 nM Mibolerone alone or in combination with Troglitazone or EPI-001 for 48 hrs at 37°C. Explants were then removed from the sponges and lysed for western blot or qRT-PCR analysis using a tissue grinder in the appropriate lysis buffer.

## Oil red O staining

Oil-red O staining was performed on 3T3-L1 cell cultures as previously described [8]. Briefly, cells were fixed in Baker's Formalin for 30 min at room temperature, followed by staining in a 60% (w/v) solution of Oil-red O (Sigma-Aldrich, Milwaukee, WI) in isopropyl alcohol for 10 min.

## pH stability studies of EPI-001 and thiol reactivity assays

### General

2-Mercaptoethanol, cysteamine, and rose bengal were purchased from Sigma-Aldrich, and reduced glutathione and tris(2-carboxyethyl)phosphine hydrochloride salt (TCEP•HCl) were purchased from Alfa Aesar. All chemicals were used without additional purification unless noted.

A solution of thiol (10 equiv.) and TCEP•HCl (100 µL of a 0.5 M DMSO stock soln.) in 1x aqueous PBS (5 mL) was adjusted to the desired pH (2.4, 7.4, or 9.4) using either aqueous HCl (6 M) or aqueous NaOH (6 M) as determined by a pH meter (Thermo Scientific Orion 3 Star). Reduced l-glutathione, 2-mercaptoethanol, and cysteamine [9], were used as thiols in this assay. To a solution of the appropriate individual thiol (170 µL) was added either EPI-001 or compound 2 (1 equiv.; both compounds were solubilized as 50 mM DMSO stock solutions). The total composition of DMSO did not exceed 7.5% in any experiment and the ratio of thiol to EPI-001/compound 2 was 10:1. *Note: This order of addition is key to achieving the appropriate pH environment for the reaction as the addition of reduced l-glutathione and TCEP acidify neutral solutions*

*and cysteamine basifies neutral solutions*. Aliquots of reactions were analyzed immediately (t ~ 30 min) following initial mixing by reverse phase HPLC and LC-MS. Reactions were gently shaken at 37°C for 12 h, then analyzed again using HPLC and LC-MS. Reactions were conducted in 2 mL clear screw cap glass vials (Agilent Technologies). *Note: Thirty minutes was the earliest time point that could be collected after adding EPI-001, mixing, and injecting an aliquot onto the HPLC*. The HPLC analytical method (Zorbax SB-C18 4.6 × 150 mm, 3.5 µm column, Agilent Technologies; flow rate = 1.0 mL/min) involved isocratic 10% $CH_3CN$ in 0.1% (v/v) aqueous $CF_3CO_2H$ (0 to 2 min), followed by linear gradients of 10–85% $CH_3CN$ (2–24 min) and 85%-95% $CH_3CN$ (24–26 min). LC-MS analyses were performed on an Agilent 1100 series instrument equipped with an Agilent MSD SL Ion Trap mass spectrometer (positive-ion mode) and a Zorbax SB-C18 column (0.5 × 150 mm, 5 µm, Agilent Technologies). The analysis method (15 µL/min flow rate) involved isocratic 10% MeCN (containing 0.1% TFA) in $ddH_2O$ (containing 0.1% $HCO_2H$; 0 to 2 mins) followed by a linear gradient of 10% to 90% MeCN (containing 0.1% TFA) in $ddH_2O$ (containing 0.1% $HCO_2H$; 2 to 24 mins), and isocratic 90% MeCN (containing 0.1% TFA) in $ddH_2O$ (containing 0.1% $HCO_2H$; 24–26 mins). The column was heated to 40°C. Wavelength monitored = 254 nm for all experiments unless otherwise noted. LC-MS analysis was performed on crude reaction mixtures. To quantify the amount of parent compound remaining, the area under the curve (AUC) of the parent compound was divided by the AUC of an internal standard. Rose bengal (7.5 µM) was used as the internal standard and was added immediately before HPLC analysis Experiments were performed in triplicate and values shown are the mean ± standard deviation (calculated in Microsoft Excel). Calibration curves to normalize for injection variances during HPLC analysis were generated for each compound (Supplementary Figure S20B). For both compounds, $R^2 > 0.99$.

## REFERENCES

1. Sadar MD, Mawji NR, Wang J, Anderson RJ, Williams DE, Leblanc M. Patent publication WO 2010000066 A1. 2010; January 7, 2010 International application PCT/CA2009/000902.
2. Anderson RJ, Mawji NR, Wang J, Wang G, Haile S, Myung J-K, Watt K, Tam T, Yang YC, Bañuelos CA, Williams DE, McEwan IJ, Wang Y, Sadar MD. Cancer Cell. 2010; 17:535.
3. Rauter W, Dickinger G, Zihlarz R, Lintschinger J. Z Lebensm.-Unters.-Forsch A. 1999; 208:208.
4. Lintschinger J, Rauter W. Eur. Food. Res. Technol. 2000; 211:211.
5. Chomczynski P, Sacchi N. Nature protocols. 2006; 1:581–5.
6. Centenera MM, Gillis JL, Hanson AR, Jindal S, Taylor RA, Risbridger GP, et al. Clinical Cancer Research. 2012; 18:3562.
7. Centenera MM, Raj GV, Knudsen KE, Tilley WD, Butler LM. Nature reviews Urology. 2013; 10:483.
8. Guo H, Bazuine M, Jin D, Huang MM, Cushman SW, Chen X. Endocrinology. 2013; 154:3525.
9. Olson ME, Li M, Harris RS, Harki DA. ChemMedChem. 2013; 8:112.

# SUPPLEMENTARY FIGURES AND TABLES

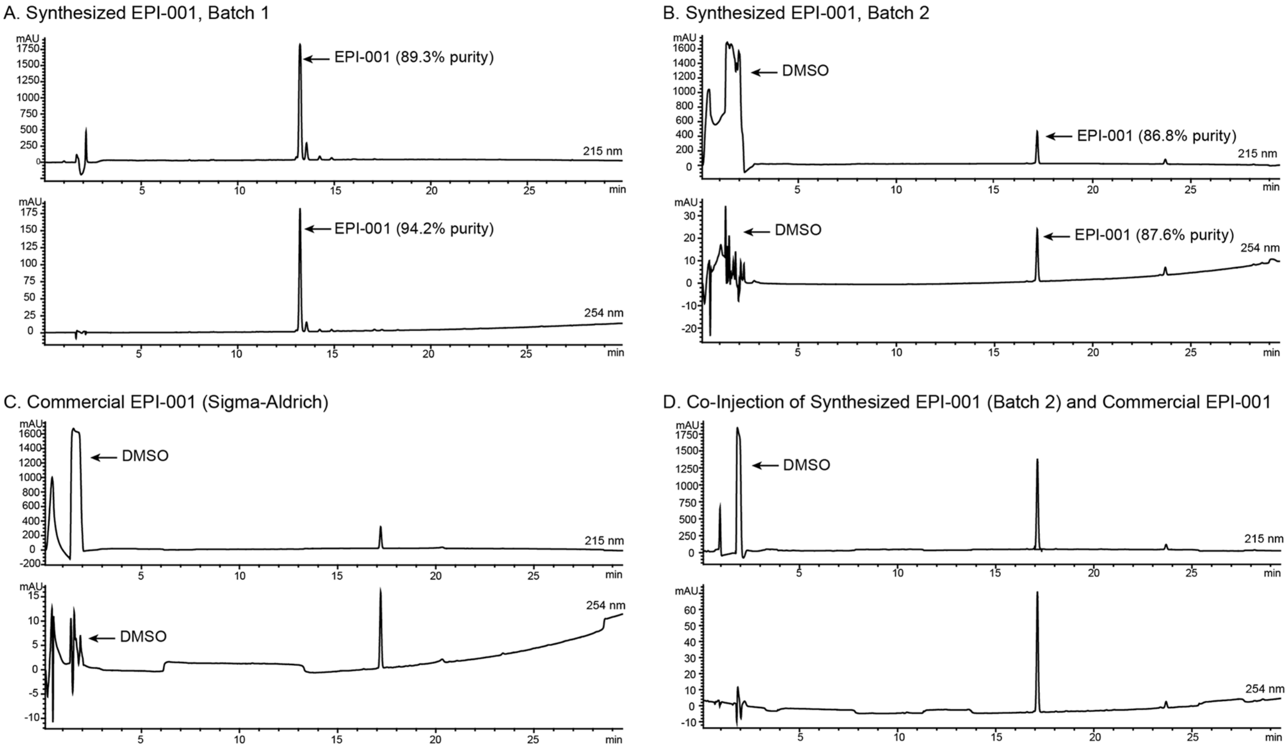


**Supplementary Figure S1: Analysis of Purity of Synthesized EPI-001 and Commercial EPI-001 (Sigma-Aldrich).** Analysis of purity of synthesized and commercial EPI-001 by reverse-phase HPLC. Solutions were monitored at 215 and 254 nm. Please see General section under Synthesis of EPI-001 for HPLC column and elution conditions. Two batches of EPI-001 were synthesized. **(A)** Batch 1 of synthesized EPI-001, which was utilized in this study. **(B)** Batch 2 of synthesized material. **(C)** Analysis of commercial EPI-001 (Sigma-Aldrich Product # 92427). **(D)** Co-injection of synthesized EPI-001 (from panel *B*) and commercial EPI-001 (panel *C*), which further demonstrates the compounds are identical. NOTE: Retention times for synthesized EPI-001, Batch 1 and Batch 2 (panels *A* and *B*) are slightly different because they were analyzed almost two years apart and HPLC column performances vary with use. $^{1}$H NMR spectra of batches 1 and 2 are identical.

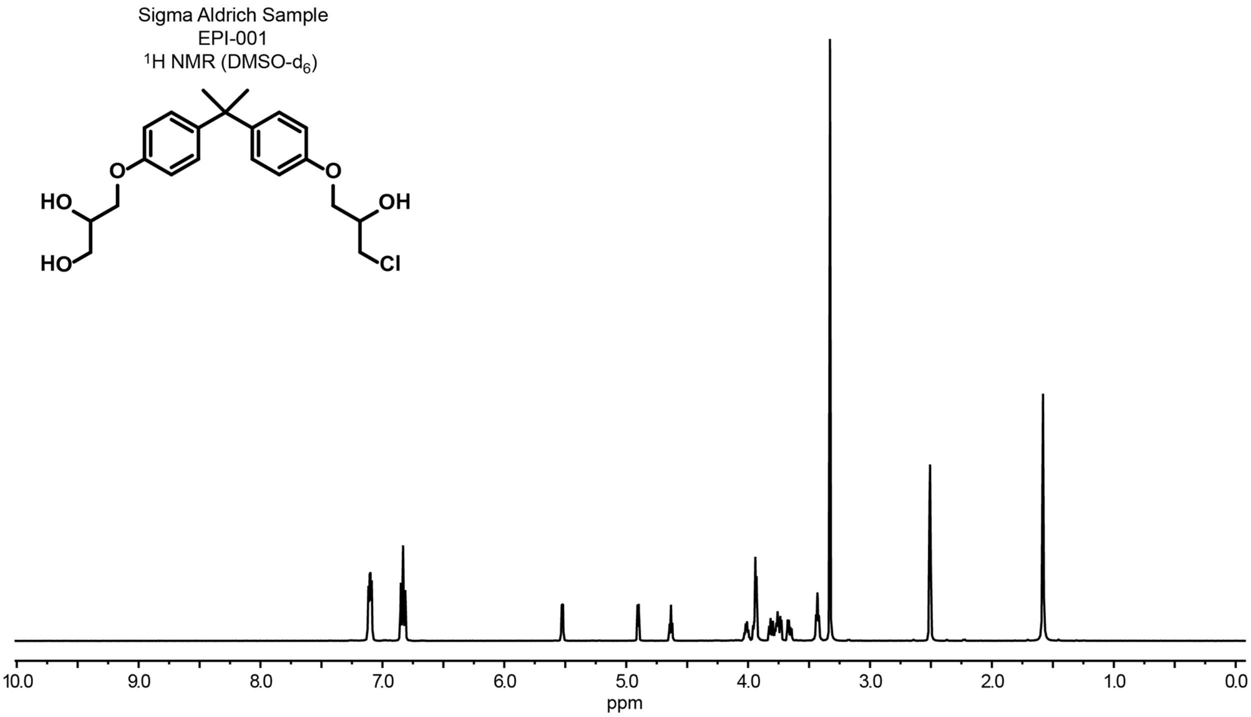
Sigma Aldrich Sample
EPI-001
$^1$H NMR (DMSO-$d_6$)
HO
HO
O
O
OH
Cl
10.0 9.0 8.0 7.0 6.0 5.0 4.0 3.0 2.0 1.0 0.0
ppm

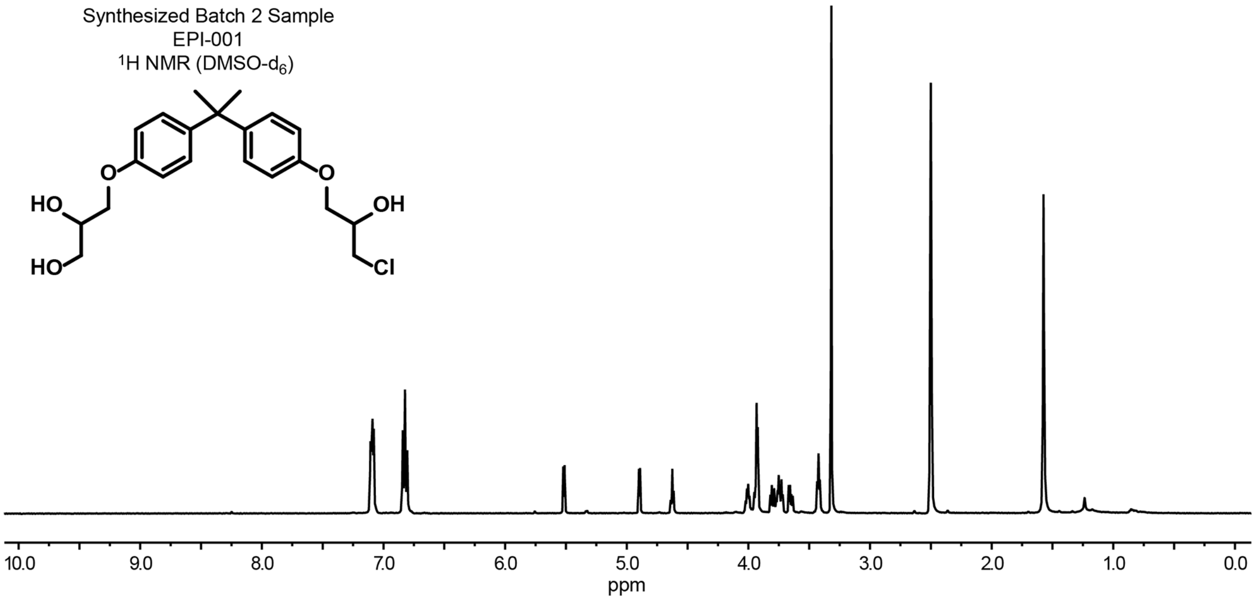
Synthesized Batch 2 Sample
EPI-001
$^1$H NMR (DMSO-$d_6$)
HO
HO
O
O
OH
Cl
10.0 9.0 8.0 7.0 6.0 5.0 4.0 3.0 2.0 1.0 0.0
ppm

*(Continued)*

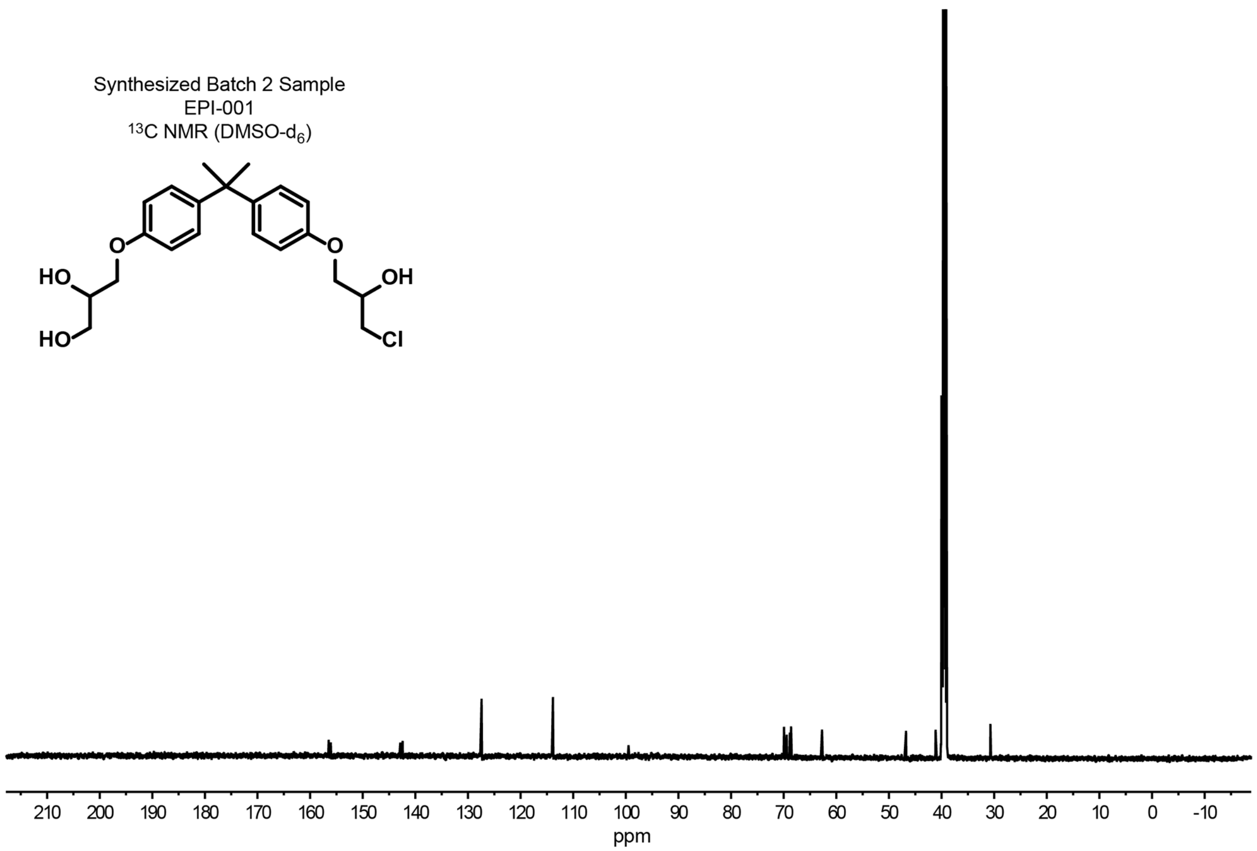
Synthesized Batch 2 Sample
EPI-001
$^{13}C$ NMR (DMSO-$d_6$)
HO
HO
O
O
OH
Cl
210 200 190 180 170 160 150 140 130 120 110 100 90 80 70 60 50 40 30 20 10 0 -10
ppm

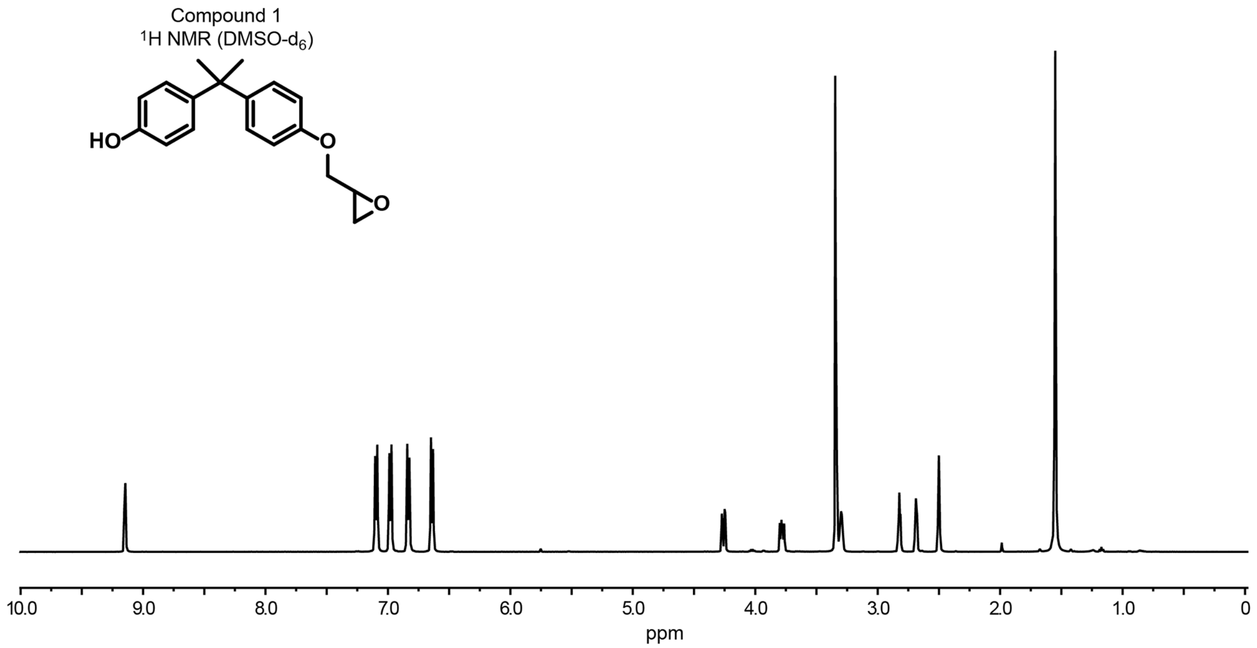
Compound 1
$^{1}H$ NMR (DMSO-$d_6$)
HO
O
O
10.0 9.0 8.0 7.0 6.0 5.0 4.0 3.0 2.0 1.0 0
ppm

(*Continued*)

Compound 1
$^{13}C$ NMR (DMSO-$d_6$)

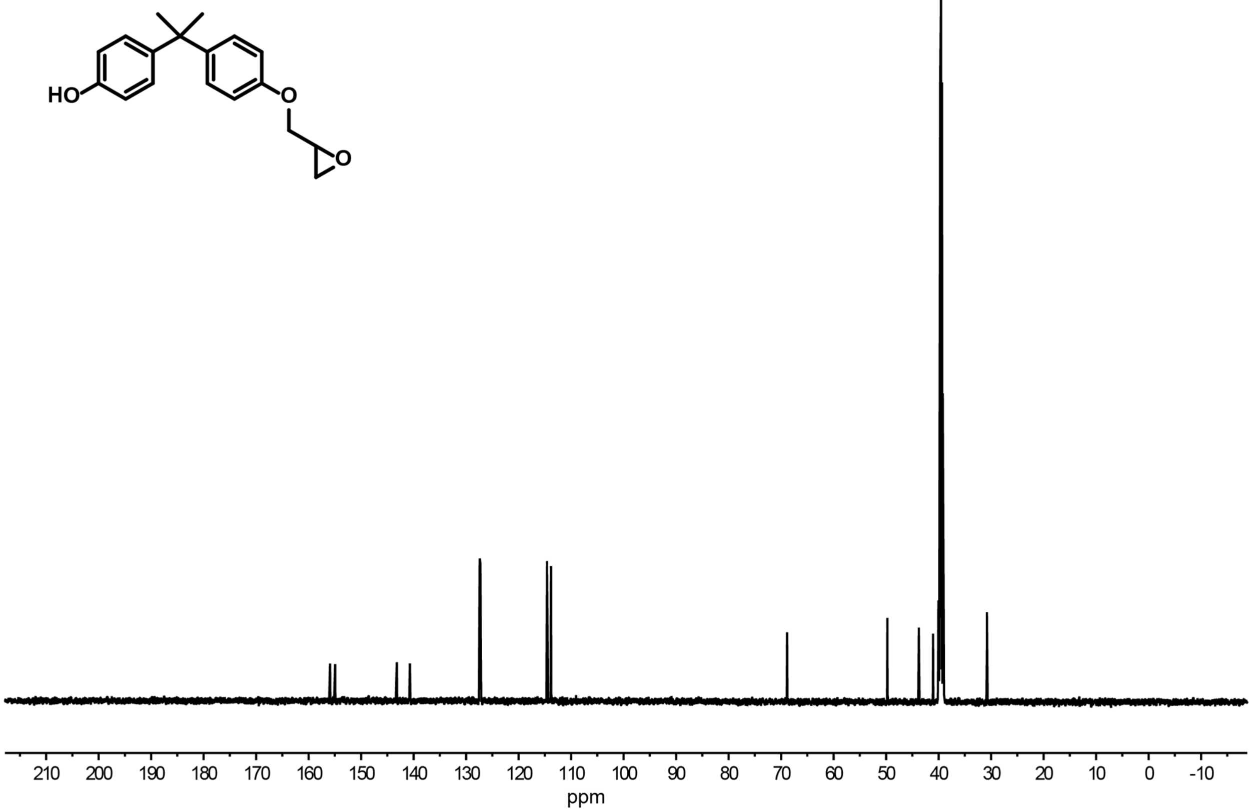


Compound 2
$^{1}H$ NMR (DMSO-$d_6$)

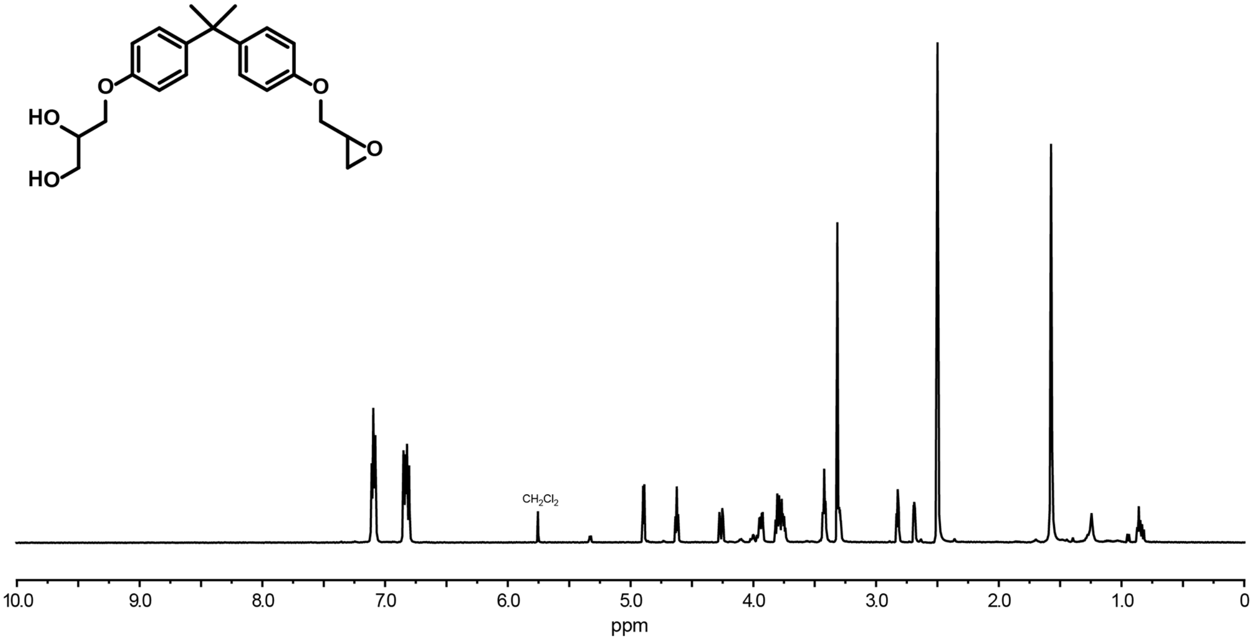


*(Continued)*

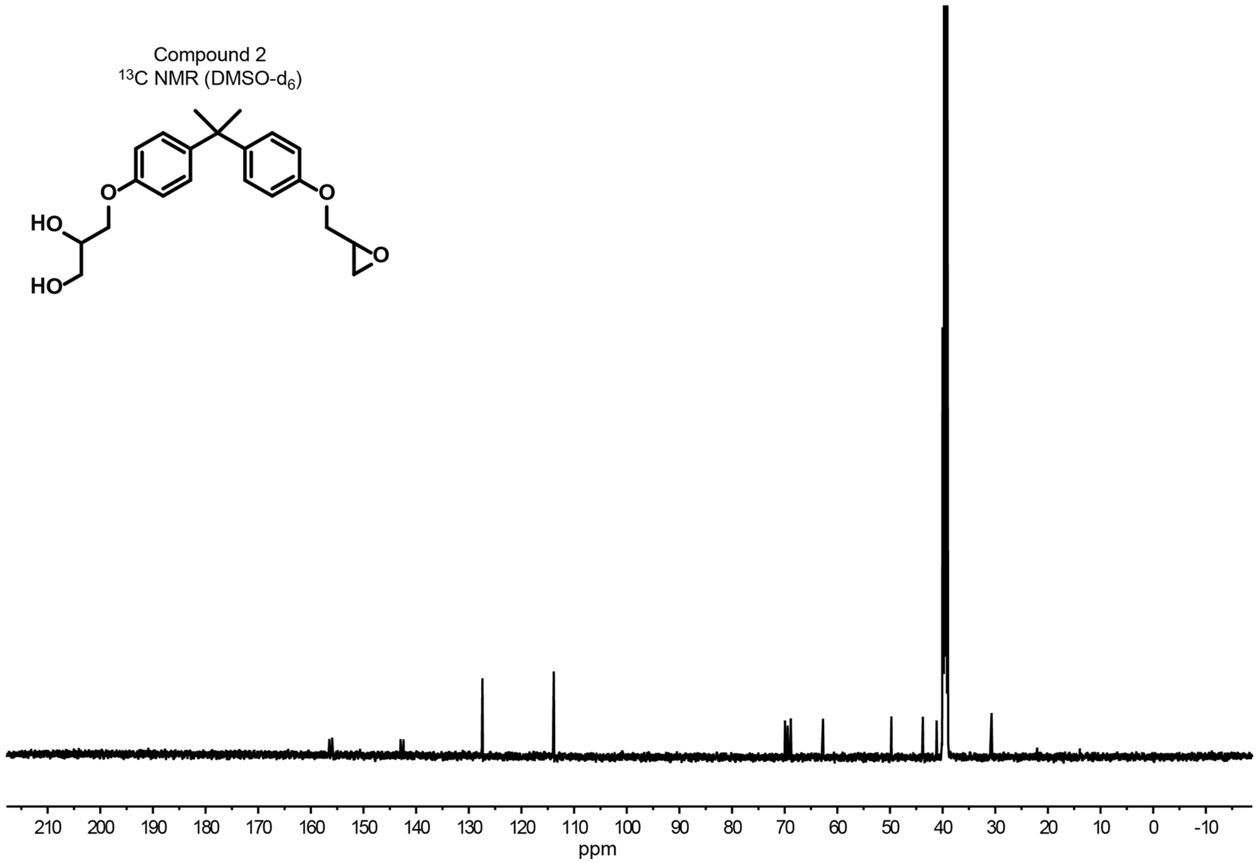


**Supplementary Figure S2: NMR Spectra of Commercial EPI-001, Synthesized EPI-001 and Reaction Intermediates.**

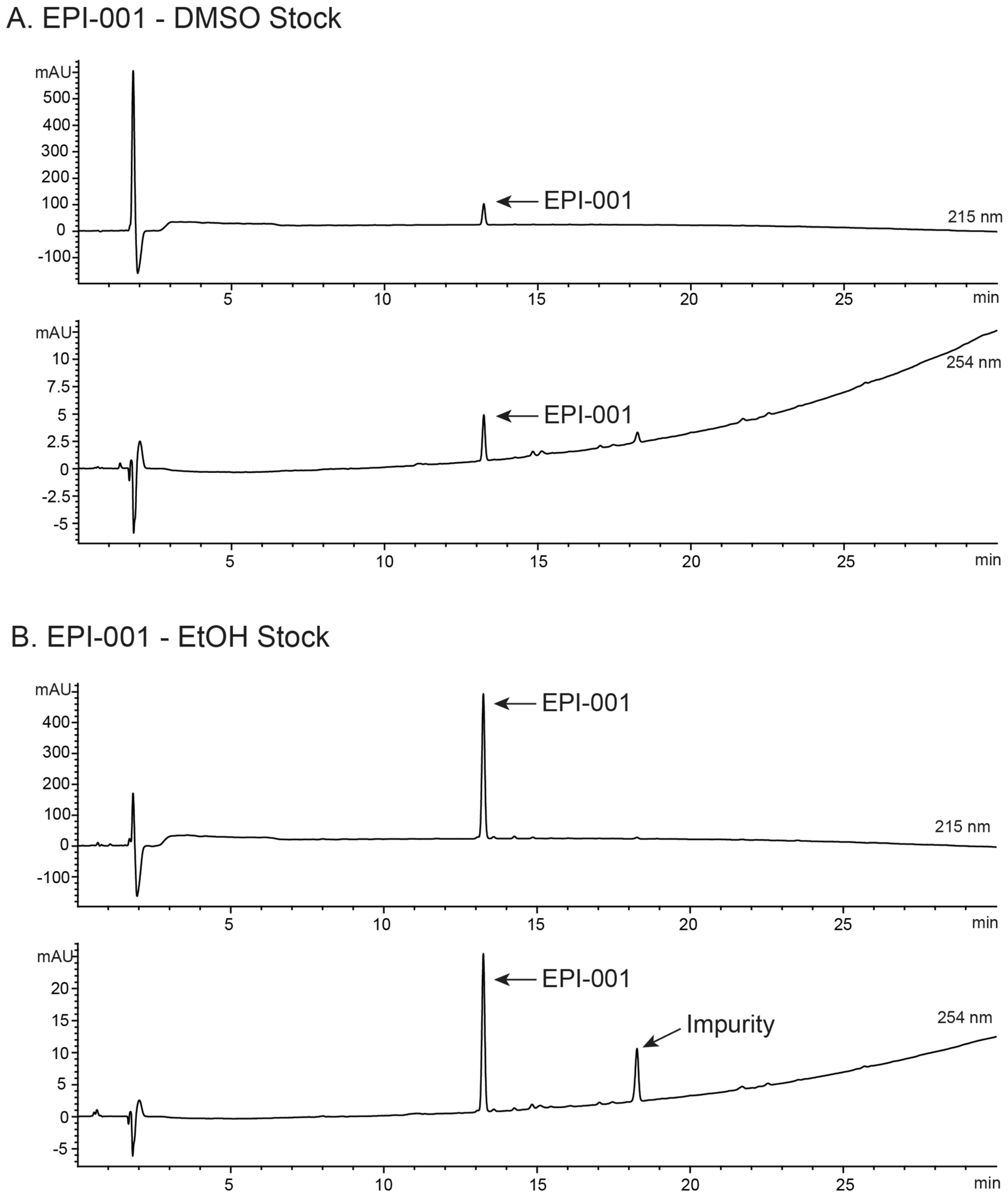


**Supplementary Figure S3: Stability of EPI-001 Stock Solutions in DMSO and Ethanol.** Analysis of purity of EPI-001 solutions prepared in either DMSO (panel A) or EtOH (panel B). Stock solutions were prepared by dissolving lyophilized drug in either EtOH or DMSO at a concentration of 100 mM overnight at 4°C. Stocks were stored at 4°C for up to six months for use in experimentation. A substantial impurity was observed in the 254 nm channel for EPI-001 solutions prepared in EtOH. Following detection of the impurity, all stock solutions were prepared in DMSO and aliquoted for storage at −20°C to prevent decomposition.

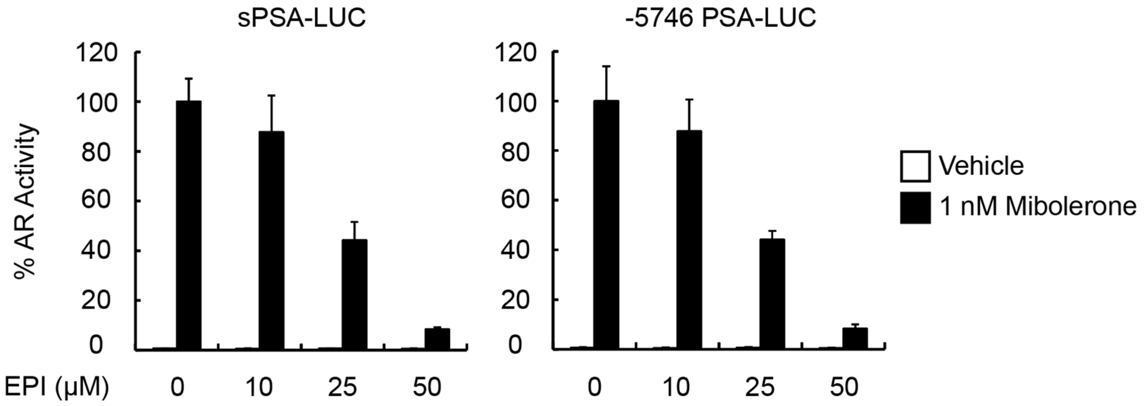


**Supplementary Figure S4: EPI-001 titration to determine maximal AR inhibition on two androgen-responsive reporters.** LNCaP cells were transfected with the indicated reporter constructs and cultured for 24 h in androgen-depleted medium. Cells were then transferred to serum-free medium supplemented with 1 nM Mibolerone or vehicle control, and treated 24 h with increasing concentrations of EPI-001 as indicated. Bars represent mean +/– SE for *n* = 6 samples from two biological replicates.

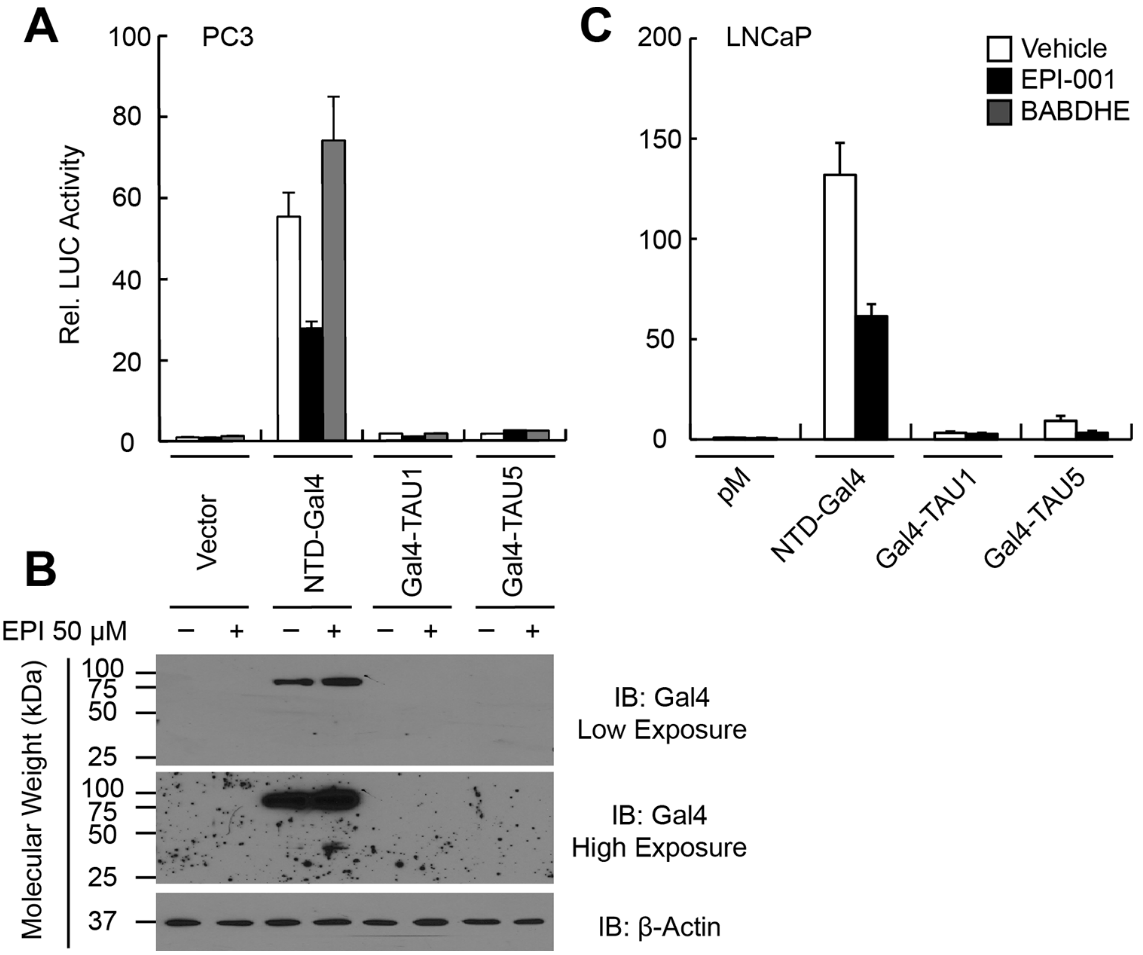


**Supplementary Figure S5: Gal4-tethered NTD domains display cell line-specific activity. (A)** PC-3 and **(C)** LNCaP cells were transfected with the indicated NTD domains tethered to Gal4 DBD (see Figure 1A for schematic) and the Gal4-responsive pG5-Luciferase reporter, then treated overnight with 50 μM EPI-001, 50 μM BABDHE, or vehicle control. **(B)** PC-3 cells were transfected with the indicated constructs corresponding to panel (A) and treated overnight with 50 μM EPI-001 or vehicle control. Lysates were subjected to western blot. Bars represent mean +/– SE for *n* = 6 samples from two separate experiments.

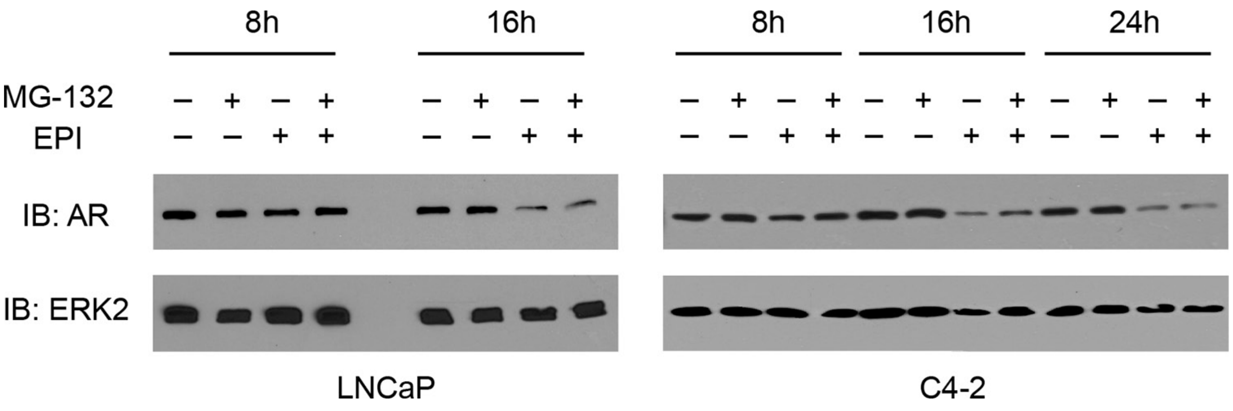


**Supplementary Figure S6: EPI-001-mediated AR protein loss occurs independently of the proteasome.** LNCaP (left) and C4-2 (Right) were serum starved overnight, then treated with 50 µM EPI-001 and/or 10 µM MG-132 as indicated. EPI-001-mediated AR protein loss occurred between 8 and 16 hours and was not reversed by proteasome inhibitor treatment.

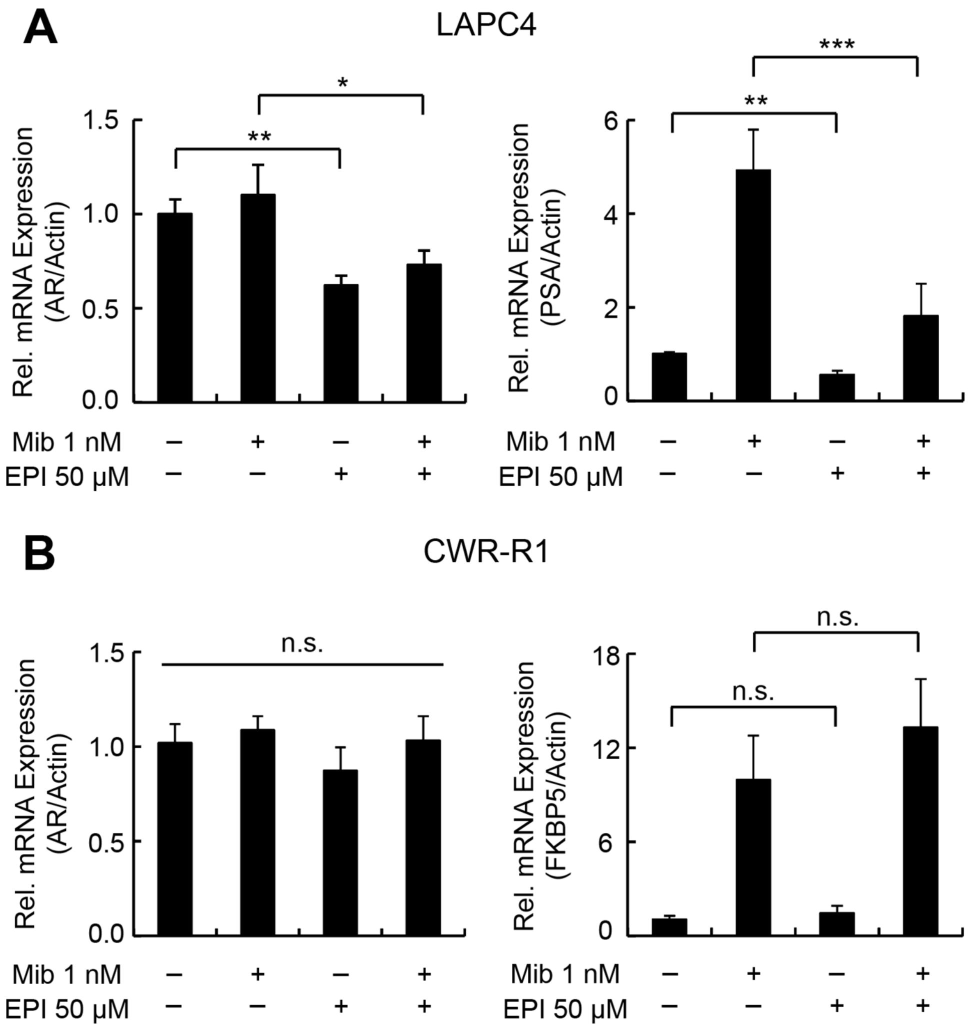


**Supplementary Figure S7: Reduced AR expression is required for EPI-001-mediated inhibition of AR activity.** LAPC4 **(A)** and CWR-R1 **(B)** cells were cultured in androgen-depleted medium for 48 hours, then treated 24 hours in serum-free medium with Mibolerone and/or EPI-001 or vehicle control as indicated. RNA extracts were prepared and subjected to qRT-PCR for total AR (left panels) and AR targets (right panels).

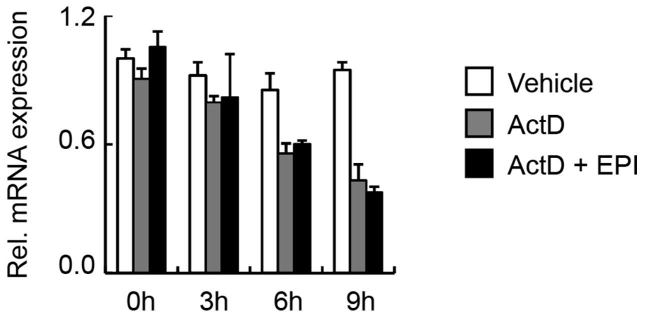


**Supplementary Figure S8: EPI-001 does not accelerate the rate of AR mRNA decay following transcriptional blockade.** LNCaP cells were serum-starved overnight, then treated with 10 µg/mL Actinomycin D +/− 50 µM EPI-001 for the indicated times. qRT-PCR analysis of AR mRNA expression relative to GAPDH was performed. Treatment with EPI-001 did not affect the rate of mRNA decay relative to Actinomycin D alone. Bars represent mean +/− SD of a triplicate experiment (*n* = 3), which was validated in a repeat triplicate experiment.

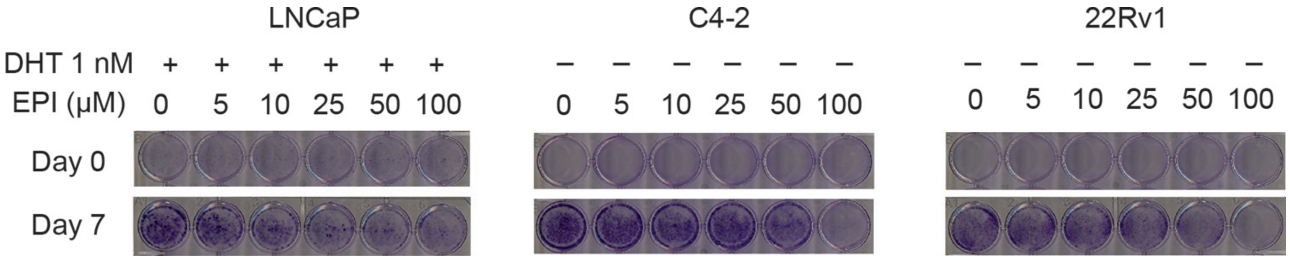


**Supplementary Figure S9: Representative scanned images of crystal violet-stained cells corresponding to Figure 3 growth assays.**

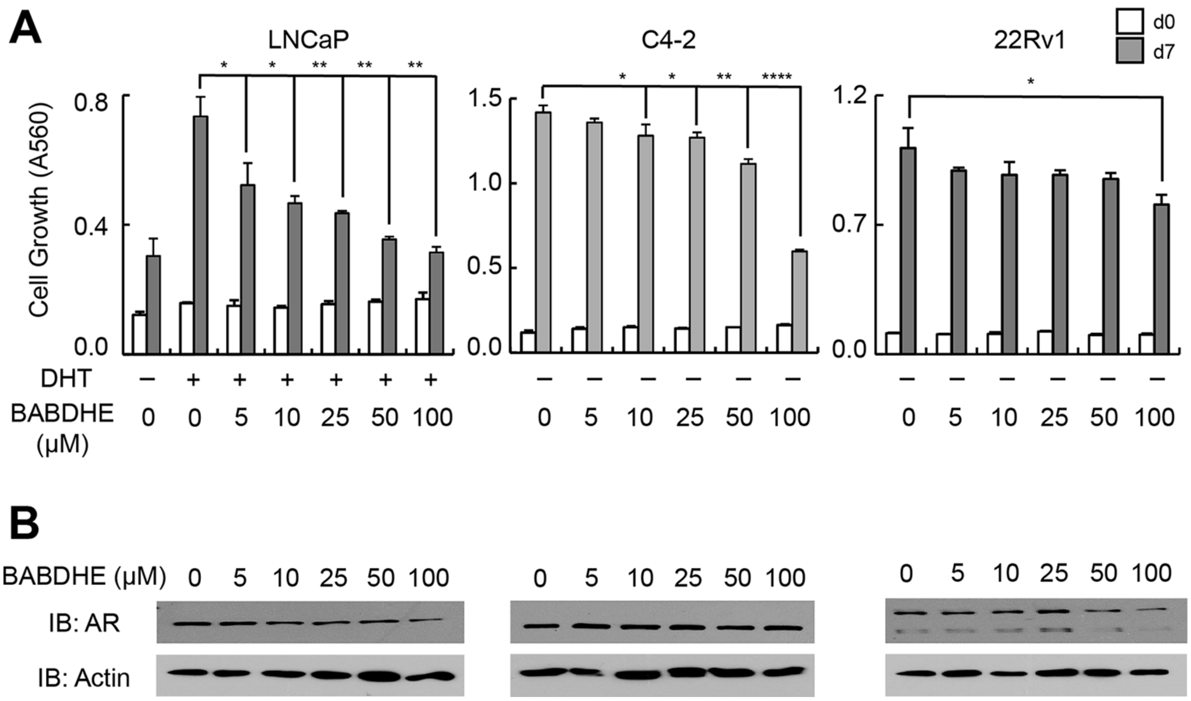


**Supplementary Figure S10: BABDHE inhibits PCa cell growth and AR expression at higher doses than EPI-001.** LNCaP, C4-2, and 22Rv1 cells were seeded as in Figure 3 and treated with 1 nM dihydrotestosterone +/− BABDHE at indicated concentrations, then subjected to crystal violet growth assays **(A)** or western blots **(B)**. Bars represent mean +/− SD for a triplicate experiment ($n$ = 3), which was validated in a repeat triplicate experiment. *$P < 0.05$; **$P < 0.01$, ****$P < 0.0001$.

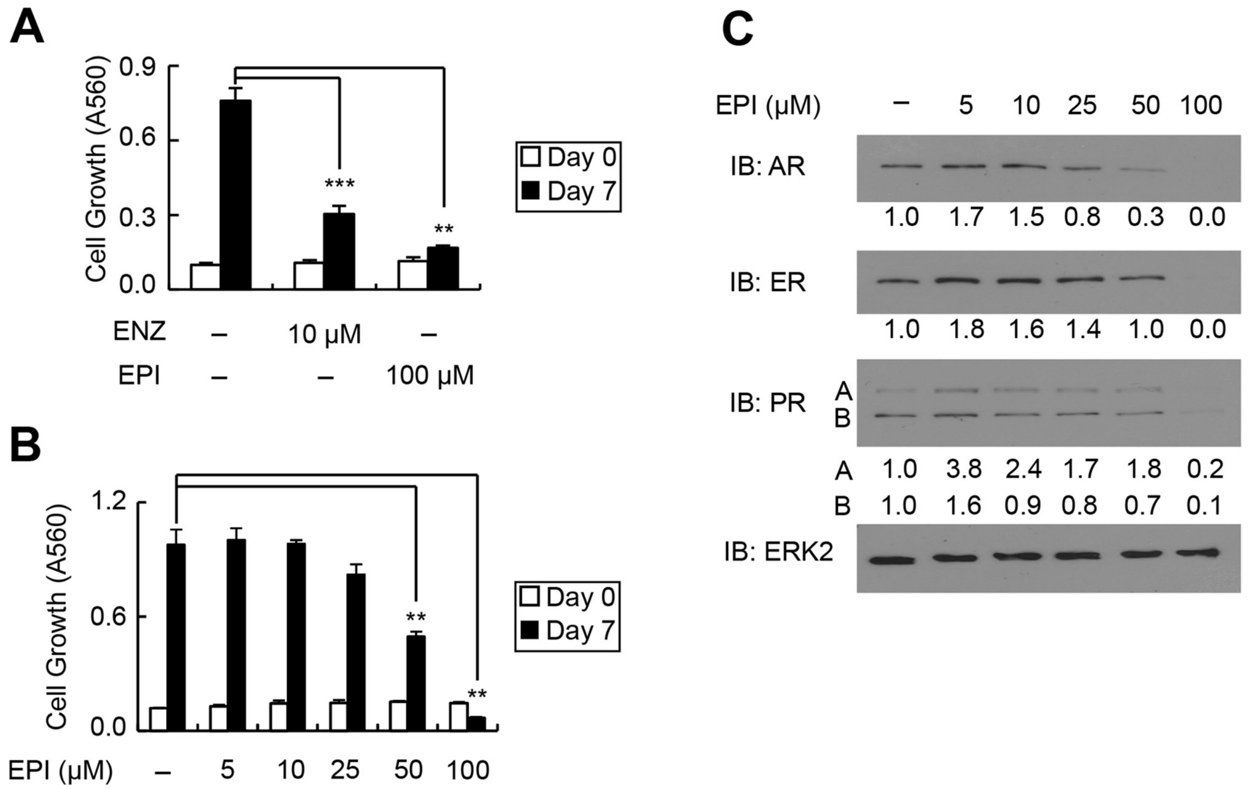


**Supplementary Figure S11: EPI-001 inhibits cell growth and alters the expression of multiple nuclear hormone receptors in T47D breast cancer cells. (A)** T47D cells were seeded in MEM +5% FBS and treated with EPI-001 or enzalutamide as indicated for 7 days, then stained with crystal violet. **(B)** Cells were seeded as in (A) and treated with increasing doses of EPI-001 for 7 days, then stained with crystal violet. **(C)** Western blot of T47D lysates treated overnight with EPI-001 in serum-free MEM as indicated. Denitometry measurements are included below their corresponding bands. Bars represent mean +/– SD from a triplicate experiment ($n = 3$), which was validated in an independent triplicate experiment. $**P < 0.01$, $***P < 0.001$.

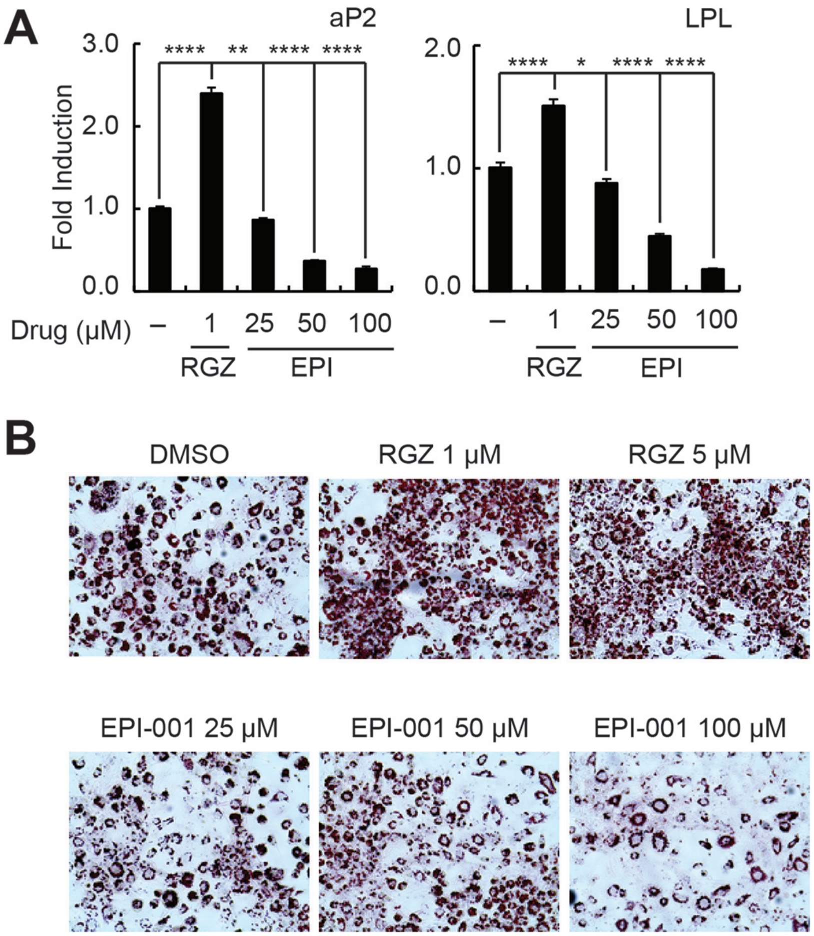


**Supplementary Figure S12: EPI-001 inhibits PPARγ activity in 3T3-L1 cells.** 3T3-L1 fibroblasts were grown to confluence and differentiated into mature adipocytes in the presence of rosiglitazone or EPI-001 as indicated. Cells were then lysed and subjected to qRT-PCR for PPARγ target genes **(A)** or stained with Oil Red O to measure lipid droplet accumulation **(B)**. Bars represent mean +/– SE of $n = 6$ samples from two independent biological replicates. $^{*}P < 0.05$; $^{**}P < 0.01$, $^{****}P < 0.0001$ relative to untreated control.

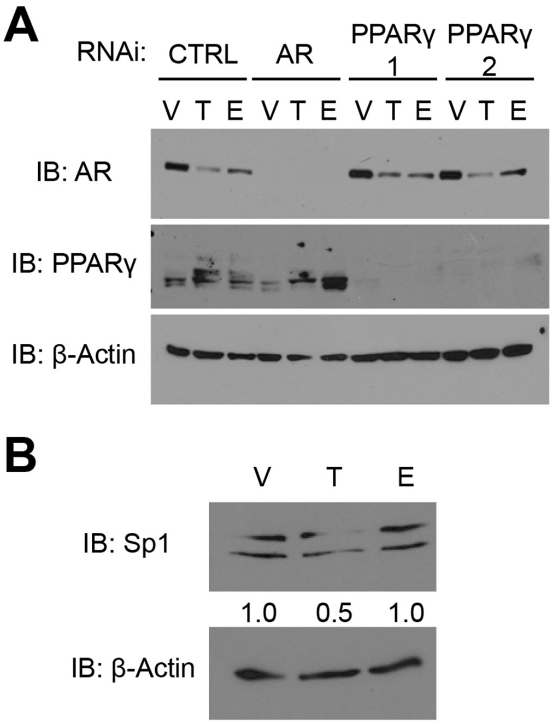


**Supplementary Figure S13: EPI-001-mediated inhibition of AR protein expression is PPARγ-independent.** **(A)** LNCaP cells were transfected with 300 pmol siRNA against AR, PPARγ, or nontargeting control and cultured in RPMI + 10% CSS for 48 hours, then treated as indicated overnight in serum-free RPMI prior to lysis for western blot for AR, PPARγ, and β-Actin (loading control). **(B)** LNCaP were seeded in RPMI +10%CSS, then treated overnight as indicated prior to lysis for western blot for Sp1 or β-Actin (loading control). V = vehicle, T = 10 µM troglitazone, E = 50 µM EPI-001.

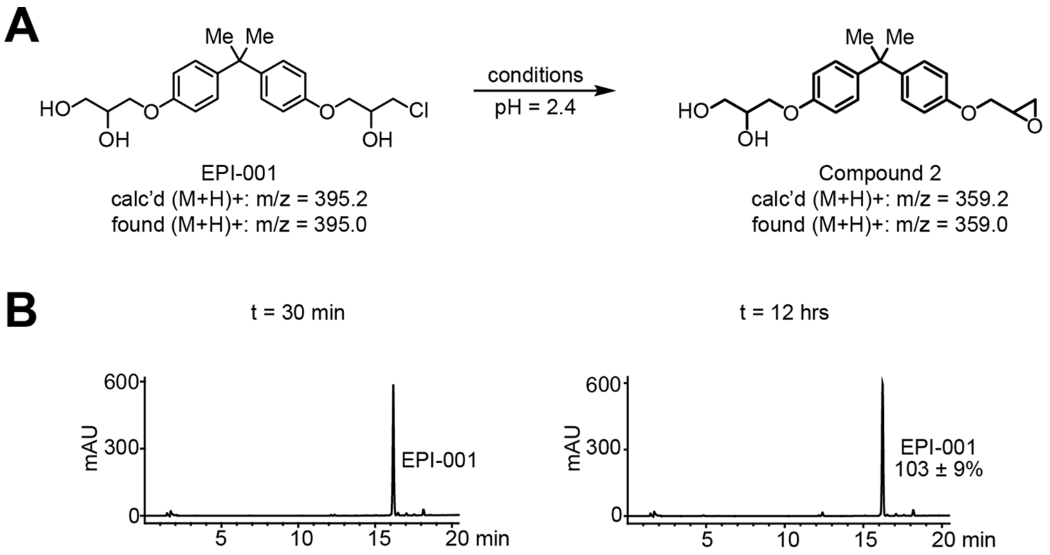


**Supplementary Figure S14: The EPI-001 chlorohydrin does not convert to an epoxide ring under acidic conditions.** **(A)** Spontaneous conversion of the EPI-001 chlorohydrin to a reactive epoxide. EPI-001 was shaken at 37°C in aq. PBS/DMSO (~10:1) at pH 2.4. The reactions were analysed by reverse-phase HPLC at t ~ 30 min and *t* = 12 h for the appearance of epoxide. **(B)** The reaction mixtures were analyzed by LC-MS confirming the absence of epoxide.

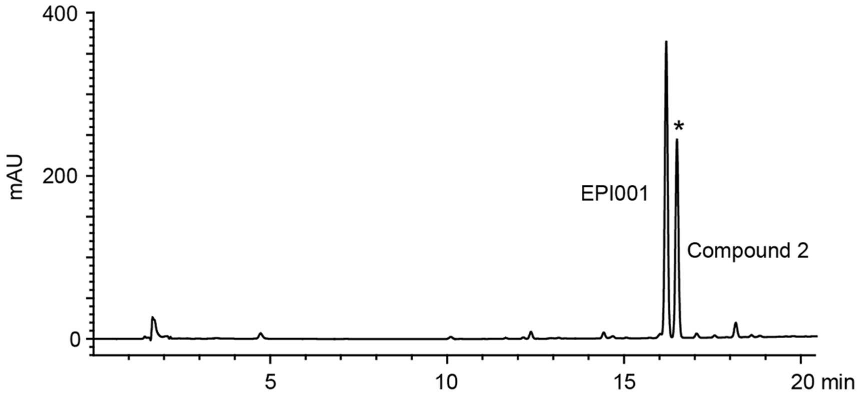


**Supplementary Figure S15: Compound 2 and the product of EPI-001 reactivity in aqueous solution are chemically identical.** A solution of EPI-001 in aq. PBS/DMSO incubated 12 h at pH = 9.4 was spiked with 12 uL of 8.5 mM Compound 2 in DMSO. The EPI-001 degradation product and the co-injected standard eluted from the column in tandem (marked with an asterisk).

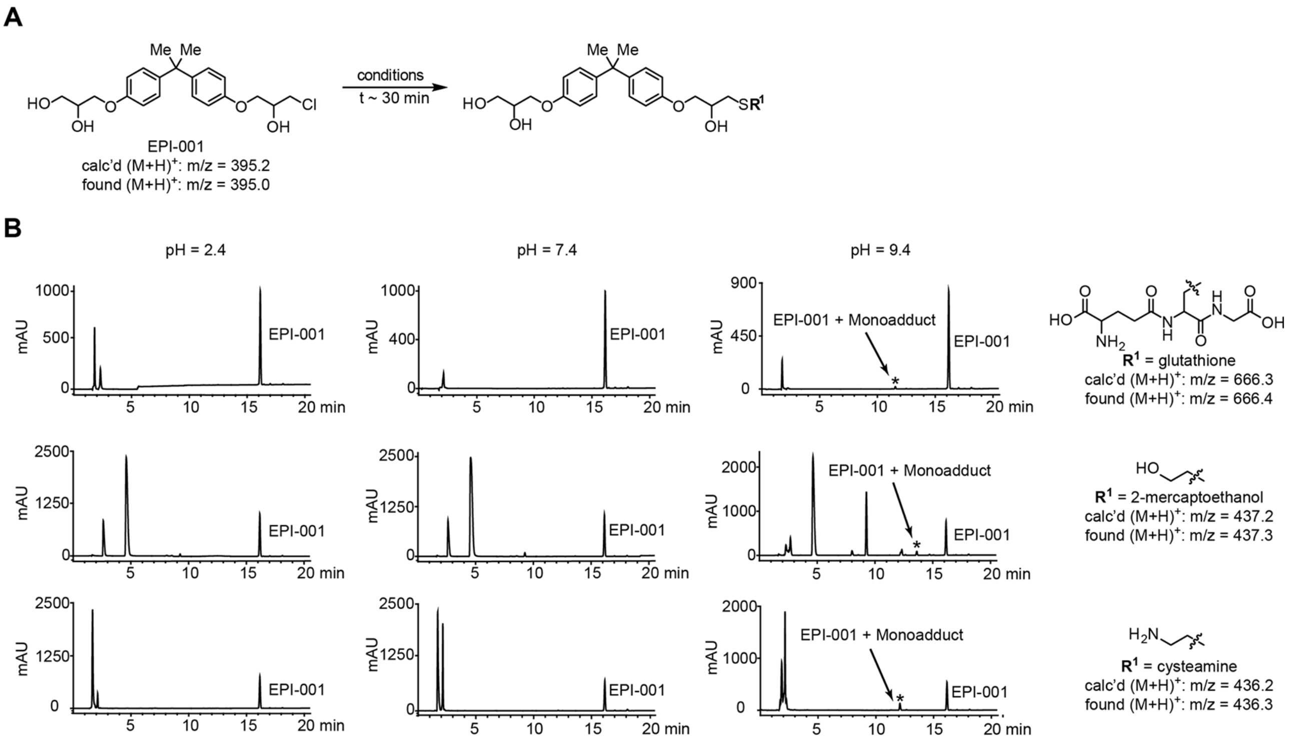


**Supplementary Figure S16: EPI-001 forms limited thiol adducts at basic pH after 30 minutes incubation.** **(A)** Solutions of EPI-001 and thiol in aq. PBS/DMSO (~10:1) at pH 2.4, 7.4, and 9.4, respectively, were shaken at 37°C. The reactivity of the following thiols with EPI-001 was investigated: reduced l-glutathione, 2-mercaptoethanol, and cysteamine. The reactions were analyzed by reverse-phase HPLC at t ~ 30 min for the appearance of thiol adducts. **(B)** At t ~ 30 min, EPI-001 adducts were only observed under strongly basic conditions (pH = 9.4). Under such conditions, EPI-001 exhibits reactivity with each of the three thiols. New products formed during the course EPI-001-thiol incubation are marked with an asterisk. Background signals collected in the absence of EPI-001 are shown in Supplementary Figure S20A. Experiments were performed in triplicate, and the identities of the monoadducts were confirmed by LC-MS.

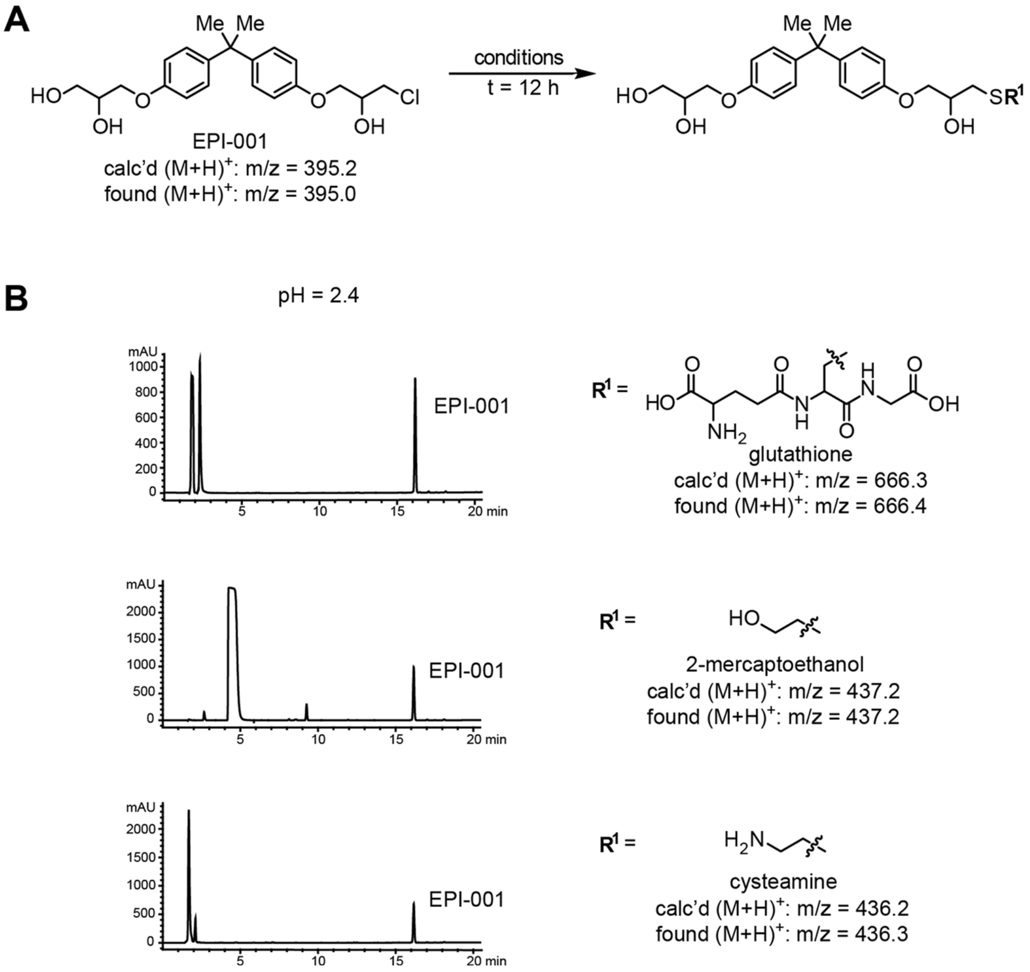


**Supplementary Figure S17: EPI-001 does not form covalent adducts with thiols under acidic conditions.** **(A)** Reaction schematic demonstrating covalent modification of reactive thiols by EPI-001. Solutions of EPI-001 and thiol in aq. PBS/DMSO (~10:1) at pH 2.4 were shaken at 37°C. The reactivity of the following thiols with EPI-001 was investigated: reduced L-glutathione, 2-mercaptoethanol, and cysteamine. The reactions were analyzed by reverse-phase HPLC at *t* = 12 h for the appearance of thiol adducts. **(B)** At *t* = 12 h, no new peaks were observed under acidic conditions.

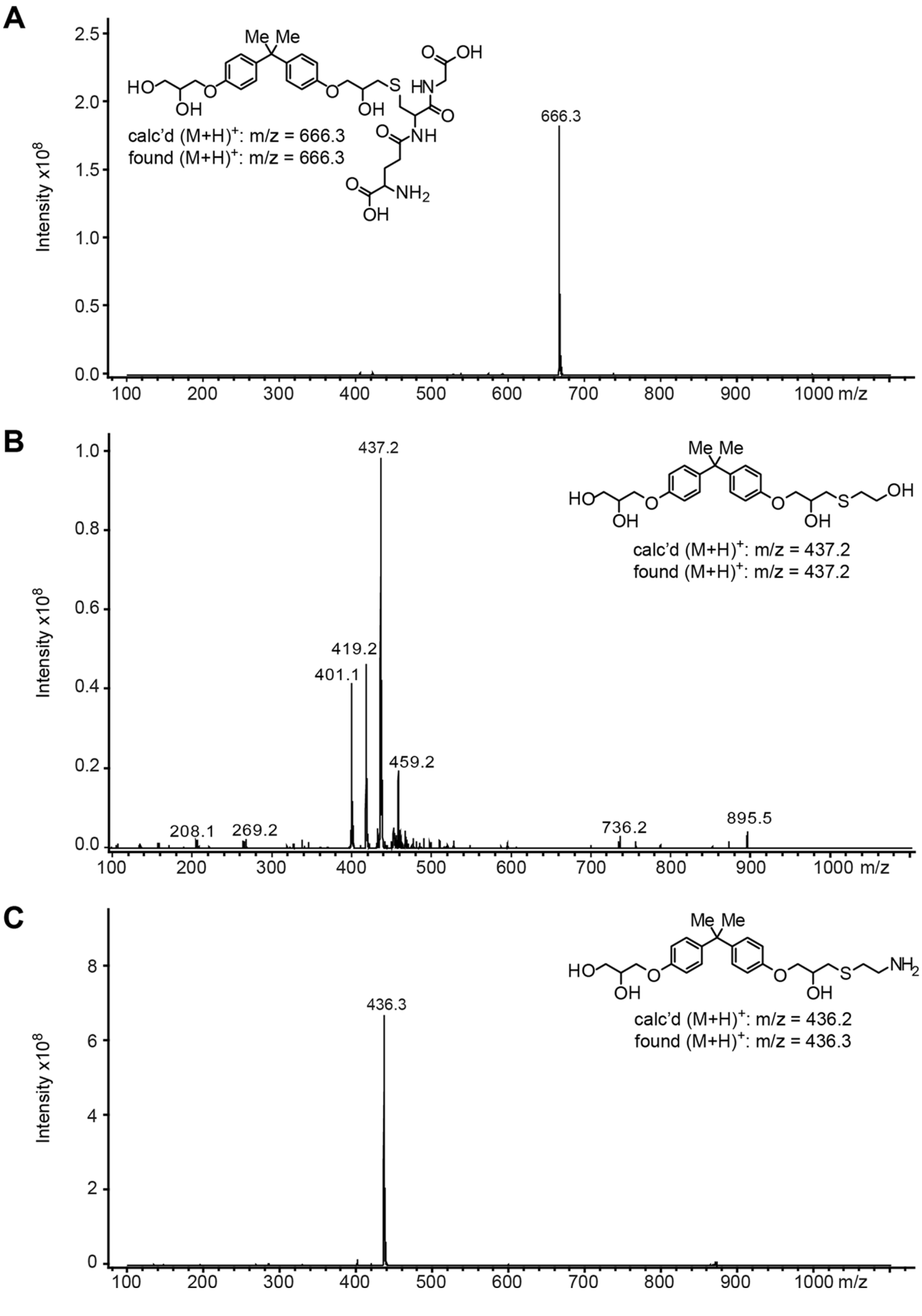


**Supplementary Figure S18: Mass spectrum of EPI-001:thiol adducts.** **(A)** Mass spectrum of the monoadduct of glutathione and EPI-001; *m*/z [M + H+] 666.3 (calc'd); 666.3 (found). **(B)** Mass spectrum of the monoadduct of 2-mercaptoethanol and EPI-001; *m*/z [M + H+] 437.2 (calc'd); 437.2 (found). **(C)** Mass spectrum of the monoadduct of cysteamine and EPI-001; *m*/z [M + H+] 436.2 (calc'd); 436.3 (found)

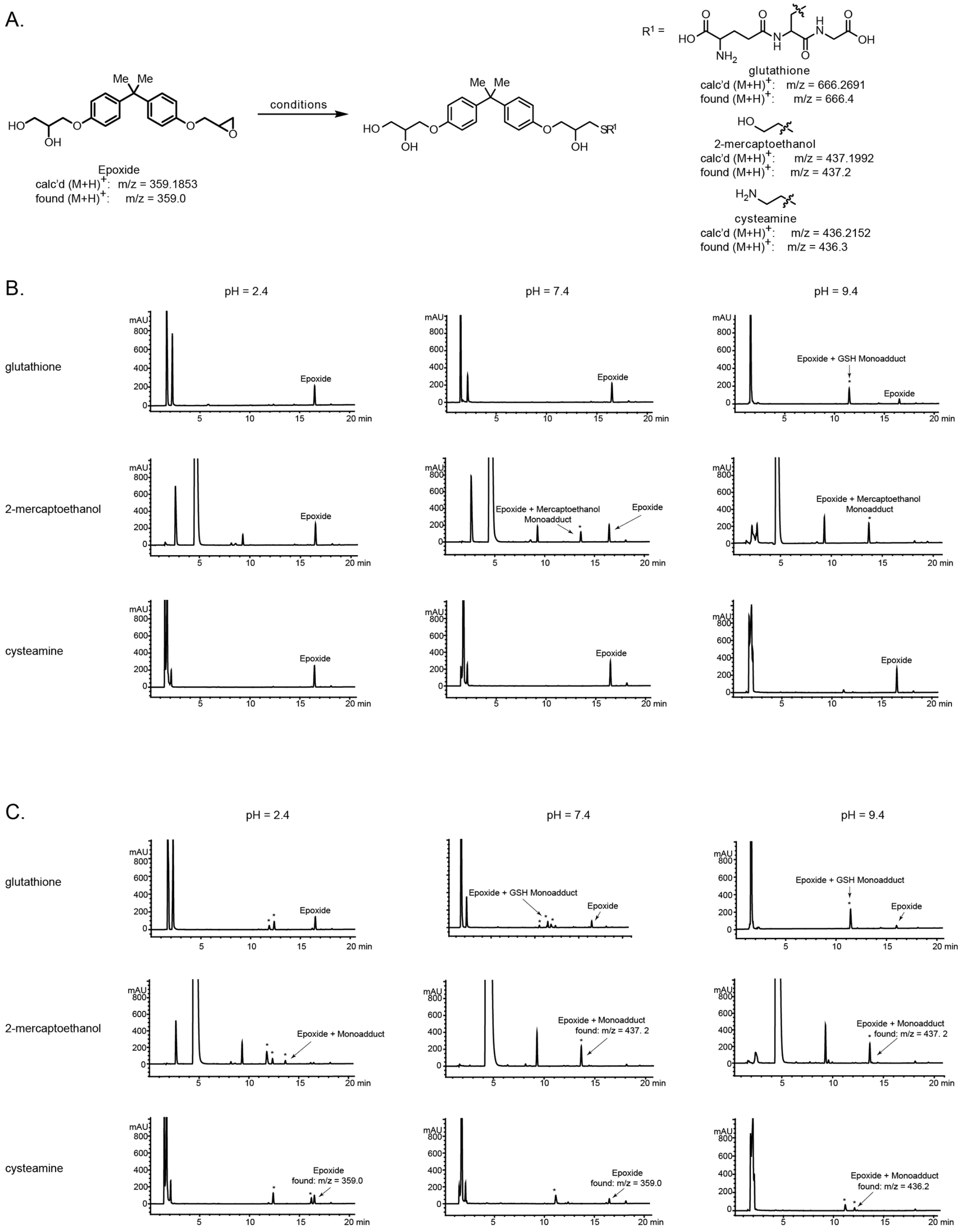


**Supplementary Figure S19: Reactivity of Compound 2. (A)** Solutions of epoxide and thiol in aq. PBS/DMSO (~10:1) at pH 2.4, 7.4, and 9.4, respectively, were shaken at 37°C. The reactivity of the following thiols with epoxide was investigated: reduced l-glutathione, 2-mercaptoethanol, and cysteamine. The reactions were analyzed by reverse-phase HPLC at t ~ 30 min and *t* = 12 h for the appearance of thiol adducts. **(B)** At t ~ 30 min, a GSH/epoxide monoadduct was observed under basic conditions (pH = 9.4) as the primary component of the reaction mixture. At pH = 7.4, a 2-mercaptoethanol monoadduct was observed. At pH = 9.4, no evidence of the epoxide remained and only the monoadduct was present. **(C)** At *t* = 12 h, a multitude of new peaks (many are thiol adducts) were observed following incubation of Compound 2 with each thiol. New products formed during the course Compound 2-thiol incubation are marked with an asterisk. Background signals collected in the absence of Compound 2 are shown in Supplementary Figure S20A. Experiments were performed in triplicate, and the identities of the explicitly assigned monoadducts were confirmed by LC-MS.

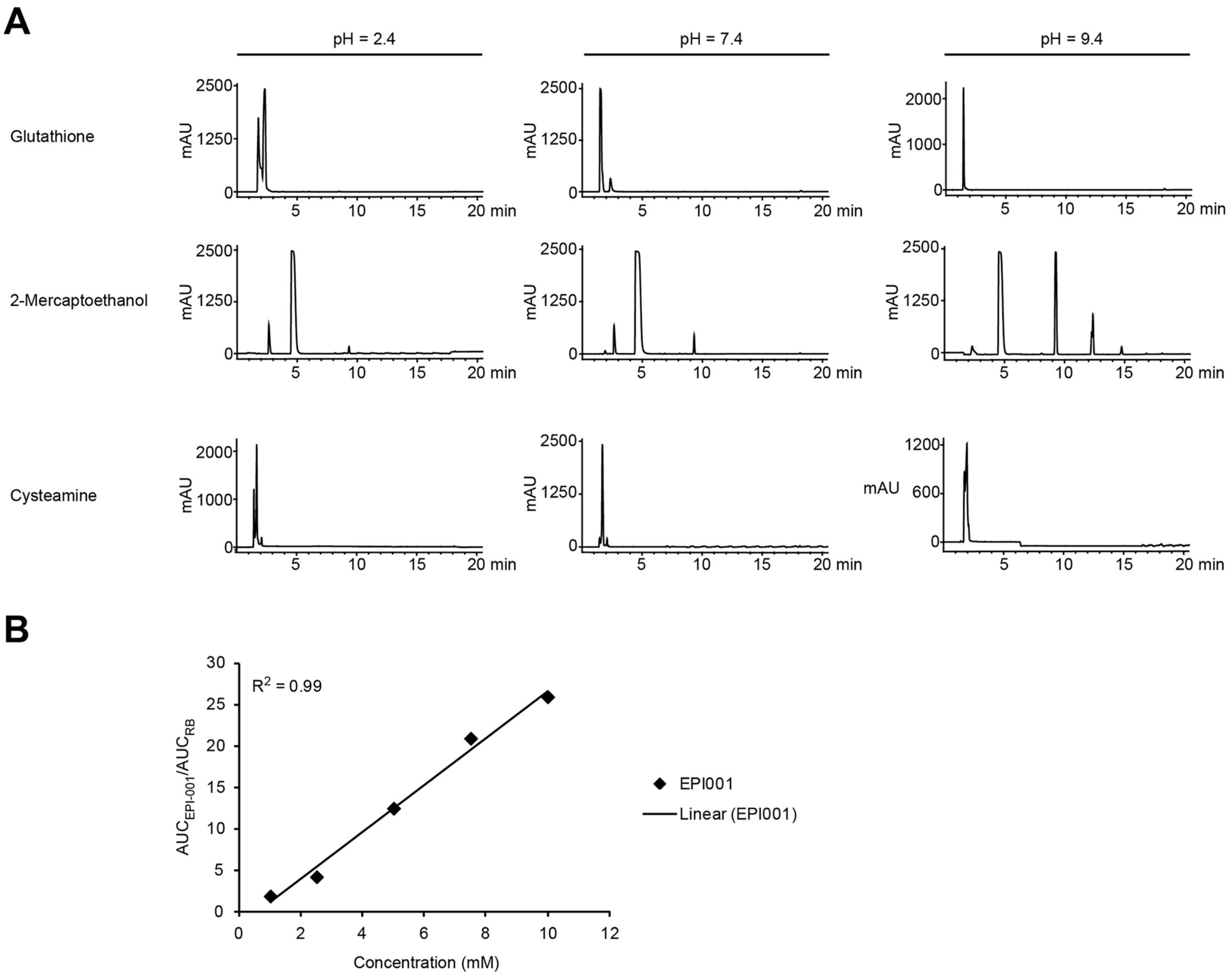


**Supplementary Figure S20: Background signals of thiols in the absence of small molecules. (A)** Solutions of the three thiols (glutathione, 2-mercaptoethanol, cysteamine) in aq. PBS/DMSO (~10:1) at pH 2.4, 7.4, and 9.4, respectively, were shaken at 37°C. The reactions were analyzed by reverse-phase HPLC at $t$ = 12 h to establish background traces. **(B)** A calibration curve was generated to normalize for injection variances during HPLC analysis.

## Supplementary Table S1: Mutagenic primer sequences for insertion of TAU1 and TAU5 into the pM vector

| Amplicon | Direction | Cut site | Sequence (5′-> 3′) |
|---|---|---|---|
| TAU1 | F | EcoR1 | CGGAATTCCGTAGAGGCCCCACAGGCTACC |
| TAU1 | R | BamH1 | CGGGATCCTCACTGGTACGCAGCTGCCTCGTCC |
| TAU5 | F | EcoR1 | CGGAATTCAGTCGCGACTACTACAACTTTCCACT |
| TAU5 | R | BamH1 | CGGGATCCTCACAGCCCCTGAGGGGGCCGAGT |

## Supplementary Table S2: Primary and secondary antibody information

| Target | Epitope | Type | Vendor | Catalog # |
|---|---|---|---|---|
| AR | N-20 | Rabbit Polyclonal | Santa Cruz | sc-816 |
| ERK2 | D-2 | Mouse Monoclonal | Santa Cruz | sc-1647 |
| Gal4 | DBD | Rabbit Polyclonal | Santa Cruz | sc-577 |
| Lamin A/C | 4C11 | Mouse Monoclonal | Cell Signaling | 4777 |
| PPAR-gamma | 81B8 | Rabbit Monoclonal | Cell Signaling | 2443 |
| beta-Actin | AC-15 | Mouse Monoclonal | Sigma | A1978 |
| HRP-Conjugated Secondary Ab | Mouse IgG | Goat | Santa Cruz | sc-2005 |
| HRP-Conjugated Secondary Ab | Rabbit IgG | Goat | Santa Cruz | sc-2004 |

## Supplementary Table S3: qPCR primer sequences

| Gene | Species | Direction | Sequence (5′ -> 3′) |
|---|---|---|---|
| AR Exon1 | Human | F | TGG ATG GAT AGC TAC TCC GG |
| AR Intron 1 | Human | R | TTT ACC CTG CTG AGC TCT CC |
| AR Exon2 | Human | R | CCC AGA AGC TTC ATC TCC AC |
| PSA | Human | F | AGG CCT TCC CTG TAC ACC AA |
| PSA | Human | R | GTC TTG GCC TGG TCA TTT CC |
| hK2 | Human | F | CTG TCA GAG CCT GCC AAG AT |
| hK2 | Human | R | GCA AGA ACT CCT CTG GTT CG |
| TMPRSS2 | Human | F | CTG CCA AGG TGC TTC TCA TT |
| TMPRSS2 | Human | R | CTG TCA CCC TGG CAA GAA TC |
| FKBP5 | Human | F | AGG AGG GAA GAG TCC CAG TG |
| FKBP5 | Human | R | TGG GAA GCT ACT GGT TTT GC |
| CIDEC | Human | F | ATT GAT GTG GCC CGT GTA ACG |
| CIDEC | Human | R | CAG CAG TGC AGA TCA TAG GAA A |
| PDK4 | Human | F | AGA GGT GGA GCA TTT CTC GC |
| PDK4 | Human | R | ATG TTG GCG AGT CTC ACA GG |
| Actin | Human | F | ATG CAG AAA GAG ATC ACC GC |
| Actin | Human | R | ACA TCTGCT GGA AGG TGG AC |
| GAPDH | Human | F | GAA GGT GAA GGT CGG AGT C |
| GAPDH | Human | R | GAG GAT GGT GAT GGG ATT TC |
| TBP | Mouse | F | GAA GAA CAA TCC AGA CTA GCA GCA |
| TBP | Mouse | R | CCT TAT AGG GAA CTT CAC ATC ACA G |
| aP2 (FABP4) | Mouse | F | GAT GAA ATC ACC GCA GAC GAC A |
| aP2 (FABP4) | Mouse | R | ATT GTG GTC GAC TTT CCA TCC C |
| LPL | Mouse | F | TGA GAA AGG GCT CTG CCT GA |
| LPL | Mouse | R | GGG CAT CTG AGA GCG AGT CTT |
